# Spectral Features Differentiate Aging-Induced Changes in Parchment—A Combined Approach of UV/VIS, µ-ATR/FTIR and µ-Raman Spectroscopy with Multivariate Data Analysis

**DOI:** 10.3390/molecules28124584

**Published:** 2023-06-06

**Authors:** Antonia Malissa, Federica Cappa, Manfred Schreiner, Martina Marchetti-Deschmann

**Affiliations:** 1 Institute of Chemical Technologies and Analytics, TU Wien, Getreidemarkt 9, A-1060 Vienna, Austria; 2 Institute of Science and Technology in Art, Academy of Fine Arts Vienna, Schillerplatz 3, A-1010 Vienna, Austria

**Keywords:** parchment degradation, collagen, lipids, early damage assessment, vibrational spectroscopy, FAMD, feature selection

## Abstract

From the moment of production, artworks are constantly exposed to changing environmental factors potentially inducing degradation. Therefore, detailed knowledge of natural degradation phenomena is essential for proper damage assessment and preservation. With special focus on written cultural heritage, we present a study on the degradation of sheep parchment employing accelerated aging with light (295–3000 nm) for one month, 30/50/80% relative humidity (RH) and 50 ppm sulfur dioxide with 30/50/80%RH for one week. UV/VIS spectroscopy detected changes in the sample surface appearance, showing browning after light-aging and increased brightness after SO_2_-aging. Band deconvolution of ATR/FTIR and Raman spectra and factor analysis of mixed data (FAMD) revealed characteristic changes of the main parchment components. Spectral features for degradation-induced structural changes of collagen and lipids turned out to be different for the employed aging parameters. All aging conditions induced denaturation (of different degrees) indicated by changes in the secondary structure of collagen. Light treatment resulted in the most pronounced changes for collagen fibrils in addition to backbone cleavage and side chain oxidations. Additional increased disorder for lipids was observed. Despite shorter exposure times, SO_2_-aging led to a weakening of protein structures induced by transitions of stabilizing disulfide bonds and side chain oxidations.

## 1. Introduction

Until the present day, numerous parchment documents have been preserved in many public and private collections, including archives, libraries and religious institutions. Among the most notable examples of these exceptionally valuable artifacts are the Dead Sea scrolls, prominently dating back to the 1st century BC, which were discovered in Qumran in the 1940s [1]. Although Egyptian drawings indicate the use of parchment already in 2500 BC [2], it primarily served as the main writing material in Europe from late antiquity to the Medieval period until the invention of paper production [1]. The production of parchment from animal skins, particularly calf, sheep and goat, involves a series of manufacturing. These steps include liming, dehairing and the removal of various skin layers, such as the subcutaneous tissue, hypodermis and complete or partial epidermis, using sharp knives. The remaining skin, mostly the dermal layer, undergoes additional finishing treatments, including thinning, shaving, bleaching/dying and polishing, before being stretched and fixed onto a frame to dry under tension [1,3,4]. Parchment’s primary structural components are fibrillar collagen type I (COL1) and, to a less extent, collagen type III (COL3), depending on its origin. Collagen is generally organized in a hierarchical structure. Three polypeptide chains with a characteristic repeating -(Gly-X-Y)_n_- motif, where X and Y often represent proline and hydroxyproline, fold into right-handed helices because of the fixed angles of the peptidyl-proline or -hydroxyproline bonds. Tropocollagen, which is approx. 300 nm long with a diameter of 1.5 nm, is stabilized by hydrogen bonds and further aligned with other assemblies in a quarter-staggered arrangement forming fibrils with a diameter of 50–200 nm. These fibrils further assemble into parallel bundles called fibers [5,6,7,8,9,10]. Collagen degradation can occur through different pathways, such as denaturation, oxidation and hydrolysis, leading to the deterioration of the collagen backbone and a complete breakdown of its ordered structure [11].

Systematic studies focusing on various aspects of parchment degradation have been conducted since the late 1990s [12], followed by several internationally recognized EU-projects (contract no. SMT4-96-2101 and EVK-CT-2001-00061) in the 2000s [13,14]. These studies have resulted in a significant number of publications demonstrating the effects of temperature, light, relative humidity (RH) and atmospheric pollutants on parchment [15,16,17,18,19,20,21]. It should be noted, however, that investigations on RH and atmospheric pollutants primarily employed dynamic approaches in combination with elevated temperature to accelerate aging [15,22]. State-of-the-art methods, such as Fourier transform infrared (FTIR), Raman spectroscopy and UV/VIS spectroscopy, have been developed for the analysis and monitoring of parchment degradation [13,23,24]. Additionally, various other approaches including differential scanning calorimetry, atomic force microscopy, thermal analysis, X-ray diffraction, chromatographic methods or scanning electron microscopy have been utilized [15,18,25,26,27,28]. Infrared and Raman spectra of parchment are predominantly characterized by bond vibration frequencies corresponding to specific amide bands: amide I (1700–1590 cm^−1^), amide II (1590–1570 cm^−1^) and amide III (1300–1200 cm^−1^). High frequency vibrations of amide A (3400–3100 cm^−1^) and amide B (3080–3050 cm^−1^) are also observed in IR-spectra. Most studies have focused envelope shapes of the amide I and II bands, the areas under the absorption bands, and shifts of the band positions in IR spectra, establishing these features as spectroscopic markers for gelatinization [29]. However, more detailed information about conformational changes in secondary protein structures can be obtained by studying the second derivatives and performing band deconvolution. This approach is already well-established in skin research, particularly in relation to the amide I band, but has been rarely applied in studies on the degradation of cultural objects, with parchment being a limited factor [30,31].

The objective of this present study is to enhance our understanding of structural changes in different components of parchment—collagen, lipids and water—under the influence of light, RH and sulfur dioxide (SO_2_). Initial evaluation of surface appearance changes has been conducted through color measurements using UV/VIS spectroscopy. Moreover, detailed analysis of the amide I and II bands has been performed, including data deconvolution of the amide III band in Raman spectra and selected spectral regions in the fingerprint region below 1000 cm^−1^, which provide information about collagen crosslinks, single amino acids and structurally important disulfide bridges. Additionally, lipids and the protein–water interaction have been examined by deconvoluting the spectral region between 3600–2800 cm^−1^ in IR spectra to complement protein analysis. Subsequent factorial analysis of mixed data (FAMD), which combines classical principal component analysis (PCA) with multiple correspondence analysis (MCA), has been employed to explore the correlation between aging conditions and spectroscopic variables and identify spectroscopic features for different aging conditions.

## 2. Results and Discussion

### 2.1. Changes observed by UV/VIS Spectroscopy

**Figure 1:**
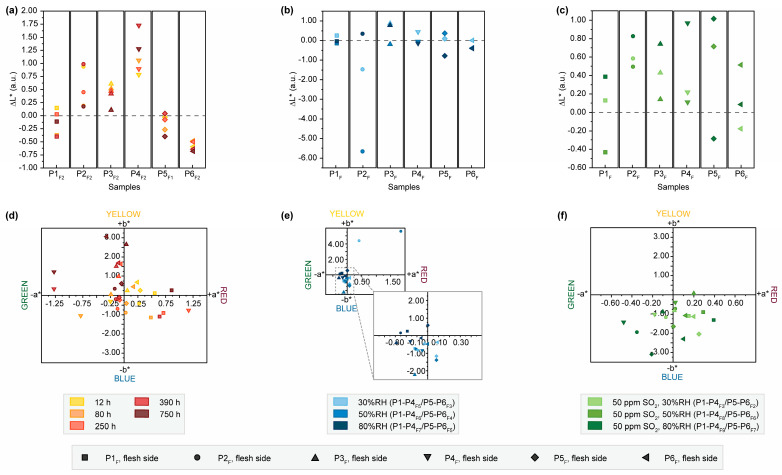
Results of colorimetric measurements after exposure of the flesh sides of P1-P6 to light, humidity and SO_2_. Changes in brightness (ΔL*) after exposure to (**a**) light, (**b**) humidity and (**c**) SO_2_. Changes of the single-color coordinates, a* on *x*-axis and b* on *y*-axis, after exposure to (**d**) light, (**e**) humidity and (**f**) SO_2_.

Directly comparing changes in color variation (ΔE*) after exposing the samples not only allows for a first evaluation of the impact of different degrading aging conditions but also provides insight into the effect of using varying concentrations of the same condition (Figure 1, Figures S2 and S3). In general, significant variations in color, perceptible to the human eye, are observed after exposure to light, RH and SO_2_. These changes are more pronounced on the hair side compared to the flesh side of the samples (see Figure S2 for details). Light-aged samples show increasing ΔE* values as the exposure time progresses [29,32], ranging from slightly perceptible (1 ≤ ΔE* ≤ 2) to easily noticeable (2 ≤ ΔE* ≤ 10) on the hair side. Exceptions to this trend are samples P4_H2_, P5_H1_ and P6_H2_, where observable changes occur only after more advanced aging. Perceptible color variations on the flesh sides are mainly observed after 80 h of exposure, except for P2_F2_ and P5_F1_. In contrast, RH-aging results in negligible color variations on the flesh side and only faint changes on the hair side. Notable exceptions are observed in sample P2 after exposure to 30 and 50%RH. Combining RH with SO_2_ leads to color variations ranging from slight to easily noticeable regardless of the exposed side.

The difference in color variation related to aging conditions and the trend of more pronounced changes on the hair side are also reflected in changes in brightness (see Figure 1a–c). A decreasing trend in brightness is observed on the flesh side of all samples with progressing exposure time and even more prominently on the hair side of P5_H1_ and P6_H2_ (Figure S4a). This differential response to light-induced aging has been reported in the literature and is likely associated with additional, chemically less stable proteinaceous components, namely reticulin and elastin, which are more concentrated on the hair side of parchment [29,32,33]. However, this trend is not observed in all samples exposed with the hair side, as an inverse correlation between exposure time and brightness is observed for P1_H2_-P4_H2_. This divergent behavior could probably be attributed to the use of chalk powder for surface treatment during production, leading to a different response to light aging. Since these parchment folios were produced over a period of ten years, this effect could also be explained by the light-induced degradation of chromophores formed during this natural and uncontrolled aging period [32]. Treatment with 80%RH results in a distinct reduction in brightness on the surface. In the literature, this effect is linked to hydrolysis-induced increase in free amino acids, contributing to the formation of brown melanoidin pigments through a Maillard reaction [24,34]. Conversely, SO_2_-aged samples show an increase in brightness with higher humidity level on both sides (Figure 1c and Figure S4c). This may be the result of surface modifications caused by the atmospheric pollutant, leading to increased brittleness, as observed in paint layers [35,36], or increasing opacity as reported in reconstituted parchment by Kern et al. [32].

The shifts in the individual color coordinates a* and b* after artificial aging provide support for the observed findings (Figure 1d–f and Figure S4d–f). Particularly, samples that underwent light aging on the flesh side exhibit shifts towards more yellow and red hues, corresponding to the darkening of the surfaces. Additionally, there is a trend of shifts towards yellow colors in the b* coordinate after exposure to 80%RH. After exposure to SO_2_, there is a shift of a* and b* to green/blue hues on both the hair and flesh sides, correlating with increased brightness.

### 2.2. Changes of Lipids and Protein Hydration Observed in m-ATR/FTIR Spectroscopy

Although the ATR-FTIR spectra of parchment are primarily dominated by the amide I-III bands, the high wavenumber region (3800–2800 cm^−1^) contains further information from CH-stretching vibrations of lipids (3000–2800 cm^−1^) or water and hydrogen bonds (3500–3000 cm^−1^) in addition to the collagen-related amide A and B bands. Figure S5 provides a comparison of the effect of light and humidity on the band shapes of these parchment components. The progressive exposure to radiation clearly leads to a decrease in the overall band intensities of lipids, amide A and B, as well as H-bond-related bands, with 250 h representing a turning point. In contrast, exposure to humidity results in an increase of the overall band intensity, directly proportional to the rising humidity content.

**Figure 2:**
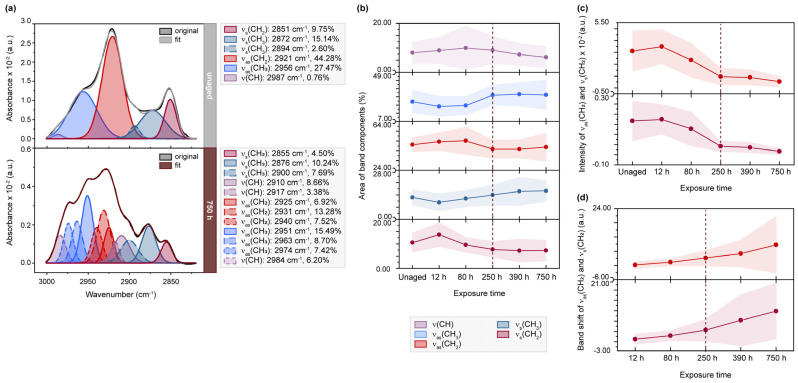
µ-ATR/FTIR spectra between 3000–2800 cm^−1^ of light-exposed flesh side of parchment. (**a**) Exemplary band deconvolution of the band envelope for lipid analysis of unaged, 250 h and 750 h exposed sample P4_F2_. (**b**) Band areas for ν(CH), ν_as_(CH_2_) and ν_s_(CH_2_), ν_as_(CH_3_) and ν_s_(CH_3_) over 750 h of exposure expressed as percentage from the band envelope area (--- 250 h turning point). (**c**) S-shaped functions observed for intensities of ν_as_(CH_2_) at approx. 2920 cm^−1^ and ν_s_(CH_2_) at approx. 2850 cm^−1^. (**d**) Blue shift with progressing exposure time of ν_as_(CH_2_) and ν_s_(CH_2_) bands (in the band envelope). (**b**–**d**) Averaged values for the flesh side of all parchment samples exposed to light.

Figure 2 presents the results of the band deconvolution, offering a more detailed understanding of the changes in overlapping bands after light exposure and different humidity levels (further details ca be found in Figures S6 and S7). An illustrative example of the band deconvolution for sample P4_F2_ shows a decrease in the band areas of components related to hydrophobic groups, from 44.28 to 27.72% for ν_as_(CH_2_) bands and from 9.75 to 4.50% for ν_s_(CH_2_) bands. This trend is also observed in the average values obtained from all samples artificially aged on the flesh side, exhibiting a significant change, particularly in the areas of ν_as_(CH_2_) after 250 h of exposure. Conversely, bands associated with hydrophilic groups, ν_as_(CH_3_) and ν_s_(CH_3_), exhibit inverse trends. The extracted band intensities of ν_as_(CH_2_) and ν_s_(CH_2_) bands at approximately 2920 cm^−1^ and 2850 cm^−1^ decrease with increasing light exposure, following an almost sigmoidal function. These changes are also reflected in the overall band shape transition of the band envelope, marked by an increasing characteristic blue shift detected in the band maxima. Similar variations in spectral parameters, such as area, intensity and band position, for the ν_as_(CH_2_) and ν_s_(CH_2_) bands have been reported for human skin treated with enhanced temperature [37,38]. The authors report an increasing band shift towards higher wavenumbers and decreasing band intensities indicating an increasing disorder in the lipid structure during phase transitions in the lipid bilayer of the stratum corneum. Since the epidermal layers are primarily removed during parchment production [39], the spectral changes result from the high dermal fat content [20,21,40] characteristic for sheepskin, as recently described by Fourneau et al. [41]. The authors reported a high greasiness and distinct fat distribution in sheepskin due to the presence of secondary follicles connected to sebaceous glands in the dermal layer, as well as the formation of a lipid layer by the secreted lipids beneath the epidermis.

Further information regarding the structural transformation of lipids is provided, complemented by details on the structural changes of proteins and protein–water interactions (Figure S7). Within the complex absorption band between 3600 and 3000 cm^−1^, there are different band components, including the amide A and B bands, which result from a Fermi resonance between NH-stretching vibrations and the first overtone of the amide II band. The results of the band deconvolution reveal a decrease in the band areas of NH-stretching within the amide A band around 3300 cm^−1^ after exposure to light (Figure S7a) and 80%RH (Figure S7b), indicating a change or progressive loss of collagen’s structural order caused by the scission of peptide bonds [42]. Previously, only band position shifts were reported to describe such changes [42]. The decrease in the amide A band component is accompanied by a comparable reduction in the sum of asymmetric (ν_as_(NH) is observed at 3343–3330 cm^−1^) and symmetric NH-vibrations (ν_s_(NH) at 3182–3162 cm^−1^), particularly after RH-aging. In comparison, the additional amide A sub-band originating from the -CH2=N- mode (around 3217 cm^−1^) remains unchanged regardless of the aging type (Figure S7) [42]. The amide B band component (around 3080 cm^−1^) is not strongly affected by the aging conditions, but its additional sub-band around 3100 cm^−1^ related to H-bonds seems to be influenced by increasing light doses, leading to a reduction in band areas (31.73 to 17.78% after 390 h of exposure) until the bands completely disappear after 750 h. Components within this broad absorption band have a direct relation to the ordered triple helix of collagen and H-bonds, with sub-bands between 3500 and 3470 cm^−1^ known to relate to intermolecular H-bonds [42]. The deconvoluted bands exhibit inverse trends in their band areas depending on the aging conditions: a consistent decrease is observed for progressing light aging, while exposure to humidity results in increasing values. The loss of hydrogen-bound water, as a result of thermal evaporation during light treatment of skin, is known to be promoting collagen degradation [42].

### 2.3. Conformational Protein Changes Observed by µ-ATR/FTIR and µ-Raman Analysis of the Vibrational Amide I, II and III Bands

The effect of artificial aging on the structural organization and stability of collagen was further assessed by analyzing the amide I and II bands in ATR-FTIR spectra and amide III bands in Raman spectra [34,37,43] While the amide I band originates from C=O stretching vibrations along the protein backbone, the amide II band represents a mixture of C-N stretching and N-H deformation vibrations. The amide III band is promoted by multiple coupling of C-N, C-H stretching and N-H bending [43]. The ATR-FTIR spectra of samples derived from P5 and P6 exhibit similar spectral characteristics for both sides of the parchment, while the spectra of samples obtained from P1–P4 are dominated by an intense absorption band at 1403 cm^−1^ on the hair side, superimposing the amide II band area (Figure S8). This absorption band was assigned to calcite, potentially originating from chalk powder residues from the production process, and therefore, these samples were excluded from the analysis of the amide II band.

**Figure 3:**
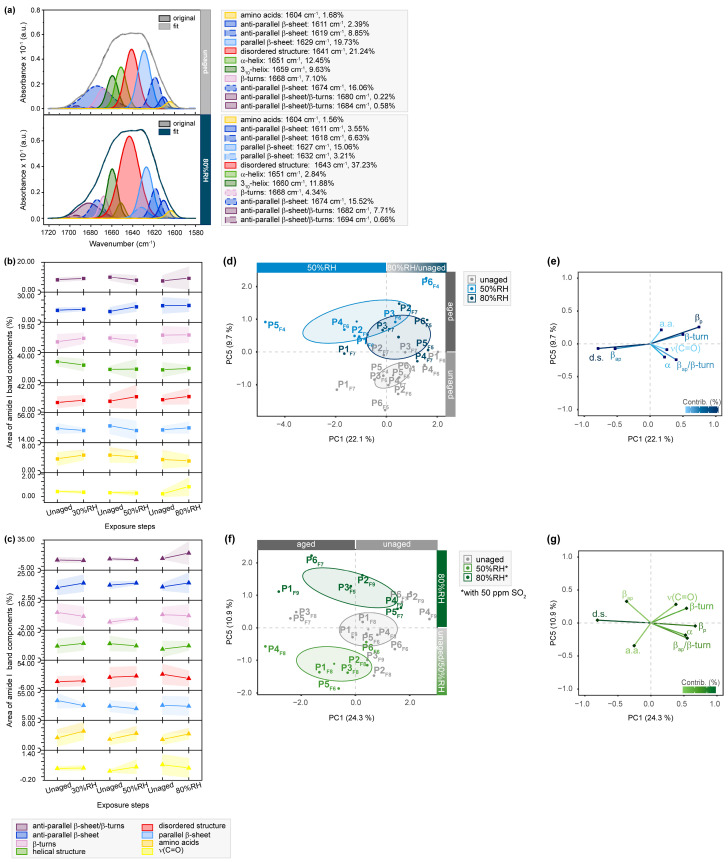
µ-ATR/FTIR spectra between 1720 and 1580 cm^−1^ of RH- and SO_2_-exposed flesh side of parchment. (**a**) Exemplary band deconvolution of the amide I band envelope of unaged and 80%RH-exposed sample P1_F7_. Band areas for amide I band components after exposure to 30%RH/50%RH/80%RH (**b**) without and (**c**) with 50 ppm SO_2_ as percentage from the band envelope. Separation of unaged and aged samples observed after RH-aging with 50 and 80%RH in (**d**) PC1/PC5 score plot. Respective contributing variables are shown in (**e**) loading plot. Separation of unaged and aged samples after SO_2_-aging with 50 and 80%RH in (**f**) PC1/PC5 score plot. Respective contributing variables are depicted in (**g**) loading plot. PCs are labelled with explained percentages of variance. Furthermore, 95% confidence ellipses are included in the score plots.

Although the exemplary amide I band deconvolution of P1_F7_ shows no apparent changes for the enveloping amide I band after exposing the sample’s flesh side to humidity, band deconvolution reveals drastic variations in the protein supra-organization after exposure to 80%RH (Figure 3). The band areas of α-helices are reduced (12.45 to 2.84%), while the area of disordered structures increases (21.24 to 37.23%), and the area fraction of anti-parallel β-sheets/β-turns is enhanced (0.22 to 7.71%). Olsztyńska-Janus et al. [37] and Cappa et al. [29] reported that intermolecular anti-parallel β-sheets are correlated with protein aggregation and denaturation resulting from thermal aging. The observed variations indicate a stepwise denaturation-like behavior of α-helix transitioning into protein aggregates before unfolding into random coils due to the hygroscopic characteristics of parchment. This behavior is especially noticeable at extreme levels of 30 and 80%RH, even in absence of enhanced temperature. The changes in β-structures are more pronounced when humidity is combined with SO_2_. Compared to humidity-exposed samples, SO_2_-induced oxidation increases band areas of amino acids. These degradation-related changes are also evident in the score plots after statistical analysis. The corresponding loading plots demonstrate that anti-parallel β-sheets and β-turns play a significant role in statistically differentiating between samples exposed to 50 and 80%RH along PC1. Changes of amino acid-assigned bands greatly contribute to the differentiation of samples exposed to SO_2_ along PC5. Although less pronounced, similar trends were observed for samples exposed with the hair sides (Figure S9). The effect of exposure to a corrosive gas compared to plain humidity is furthermore highlighted by the presence of intra-molecular parallel β-sheets within the amide II bands. Combining SO_2_ with humidity induces a reduction of the energetically less stable β-structures and a simultaneous increase in random coils, suggesting protein unfolding as an important degradation pathway.

In comparison to samples aged under humidity and SO_2_, the amide I and II bands exhibit alterations in their shapes and shifts in the maximum values of the amide I band towards higher wavenumbers and the amide II band to lower wavenumbers after light exposure (details in Figure S10). These observations have already been noted in collagen denaturation and oxidation studies [29]. The exemplary amide I band deconvolution of P1_F1_ (Figure S10a) explains the superficial changes revealing a strong decrease in α-helices at 1650 cm^−1^ and a simultaneous increase in random coils (centered at 1641 cm^−1^).

These variations are accompanied by an increasing presence of anti-parallel β-sheets and β-turns between 1680 and 1698 cm^−1^ and amino acid changes observed at 1607 cm^−1^.

Gradual changes in secondary structures are observed in the averaged band areas with increasing exposure time and are primarily responsible for distinguishing between native and aged states through factor analysis. Anti-parallel β-sheets/β-turns and amino acid residues, in particular, contribute significantly to the differentiation along PC2. These findings are supported by the band deconvolution of the amide II band in P1_F1_, which further highlights the specific amino acid vibrations represented in the amide I band originating from tyrosyl residues, known as preferred cleavage positions within the collagen molecule during oxidation [29]. The deconvolution of the amide III bands in the Raman spectra mainly confirms the previously mentioned changes in collagen conformation (Figure S11). Bands at 1340 and 1380 cm^−1^ correspond to CH_2_-stretchinng vibrations associated with proteoglycans and glycosaminoglycans connected with collagen via crosslinks [44]. An abrupt increase (12.38 to 15.06%) is observed after a light exposure of 250 h, while reduced band area ratios of these glycans are observed for all RH levels, particularly pronounced at 9.73% after exposure to 80%RH. When exposed to humidity in combination with SO_2_, mainly decreasing band areas are observed, likely due to the hydration of the water-soluble components. The results of the factor analysis emphasized the high importance of this structural feature for distinguishing between light-aged and humidity-exposed samples and their respective unaged state along PC2 and PC1 (Figures S12 and S13).

### 2.4. Changes of Single Amino Acids as Observed by µ-Raman Analysis

**Figure 4:**
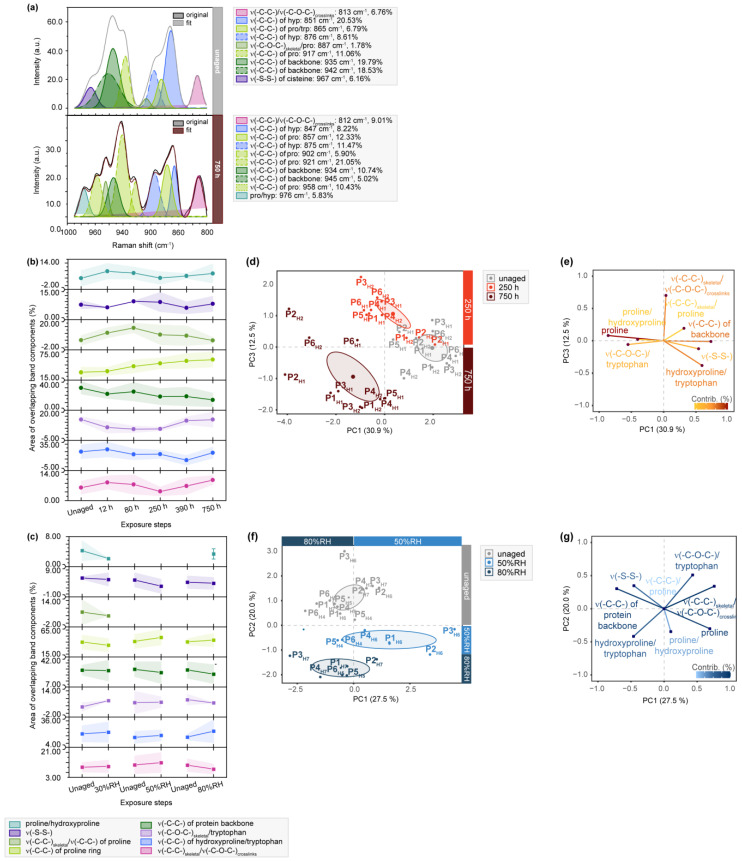
µ-Raman spectra between 1000 and 800 cm^−1^ of light- and humidity-exposed flesh side of parchment. (**a**) Exemplary band deconvolution of the band envelope of unaged and 750 h exposed sample P3_F1_. (**b**) Band areas of the respective band components after light exposure and (**c**) RH-exposure to 30%RH-80%RH as percentage from the band envelope area. Separation of unaged and aged samples observed after light-aging for 250 and 750 h in (**d**) PC1/PC3 score plot. Respective contributing variables are shown in (**e**) loading plot. Separation of unaged and aged samples after RH-aging with 50 and 80%RH in (**f**) PC1/PC2 score plot. Respective contributing variables are depicted in (**g**) loading plot. PCs are labelled with explained percentages of variance. Furthermore, 95% confidence ellipses are depicted in the score plots.

Collagen crosslinks are not only evident in amide III bands but are also accompanied by characteristic absorption signals in the C-C stretching mode region of the fingerprint region in Raman spectra (1000–800 cm^−1^). Sub-bands attributed to the protein backbone, proline and hydroxyproline, which constitute a significant portion of collagen’s amino acid backbone, are presented. Figure 4 clearly demonstrates a decrease in intensity for the enveloping band shape after light exposure. The exemplary band deconvolution of P3_H1_ highlights a significant decrease in the contribution of the protein backbone (approx. between 945 and 935 cm^−1^) after 750 h (38.32 to 15.76%), along with a strong reduction of band areas originating from a reduced number of hydroxyprolines (approx. at 850 and 875 cm^−1^).

Both observations suggest a degradation of the protein backbone and a breakdown of the helical structure [43]. Additionally, a C-O-C vibration assigned to glucosyl-galactosyl crosslinks of lysine (centered at 815 cm^−1^) [43] exhibits a slight increase (6.76 to 9.01%), particularly for the hair side of samples after light aging. As discussed earlier, RH aging induces structural change in collagen but results in inverse observations for bands originating from crosslinks. This phenomenon, as discussed by Vest et al. [13] based on physical appearance analysis, hydrothermal stability and supporting amino acid analysis, is likely linked to the formation and loss of intermolecular H-bonds and water residues still trapped in the parchment. Temperature, which increases in a light aging chamber, leads to water loss and, consequently, a reduction in intermolecular H-bonds, as observed in the presented data. Vest et al. suggest that close proximity of dry collagen molecules allows for an increasing number of crosslinks, contributing to brittleness of parchment.

**Figure 5:**
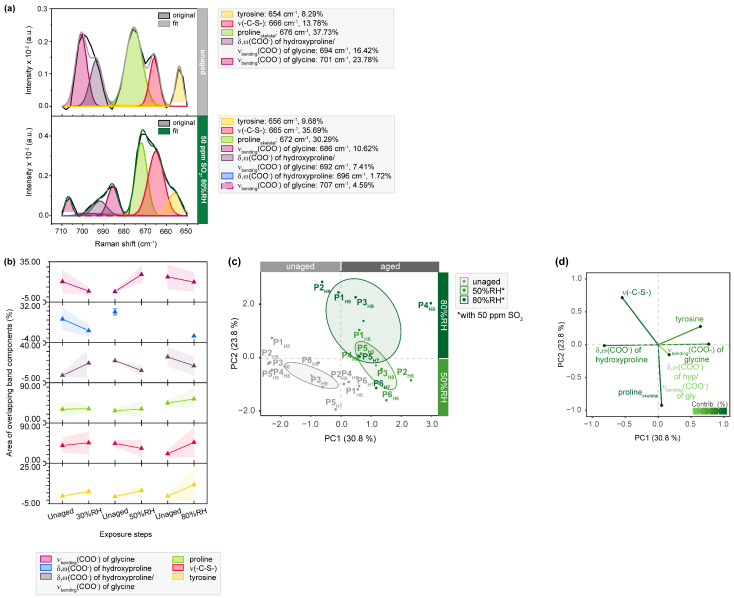
µ-Raman spectra between 710 and 650 cm^−1^ of SO_2_-aged hair sides of parchment. (**a**) Exemplary band deconvolution of the band envelope of P1_H9_ before and after exposure to 50 ppm SO_2_ and 80%RH. (**b**) Band areas of the respective band components. Separation of unaged and aged samples after aging with 50 and 80%RH observed in (**c**) PC1/PC2 score plot and contributing variables in (**d**) loading plot. PCs are labelled with explained percentages of variance. Furthermore, 95% confidence ellipses are shown in the score plot.

**Figure 6:**
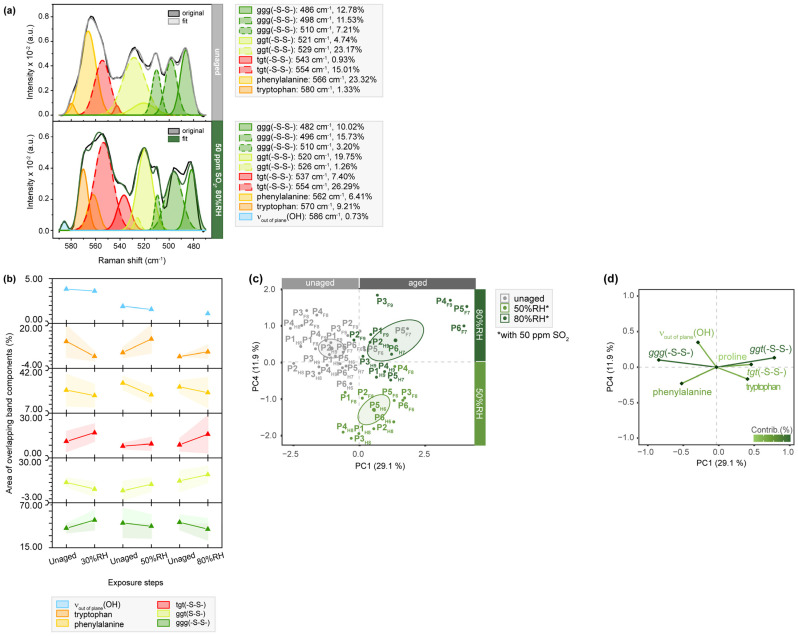
µ-Raman spectra between 590 and 470 cm^−1^ of SO_2_-aged parchment. (**a**) Exemplary band deconvolution of the band envelope of P5_F7_ before and after exposure to 50 ppm SO_2_ and 80%RH with the flesh side. (**b**) Band areas of the respective band components after exposure of the flesh sides. Separation of unaged and aged samples after aging with 50 and 80%RH observed in (**c**) PC1/PC4 score plot and contributing variables in (**d**) loading plot. PCs are labelled with explained percentages of variance. Furthermore, 95% confidence ellipses are shown in the score plot.

Spectral regions between 800 and 720 cm^−1^ (Figure S16) and 710–650 cm^−1^ (Figure 5, Figures S17 and S18) reveal additional transitions of the amino acids tyrosine, tryptophan and the sulfur-containing components methionine and cysteine. By deconvolution, increasing band area ratios for tyrosine (approx. 654 cm^−1^) are found, especially for the averaged band areas of humidity levels containing SO_2_. The oxidative stress produces electrophilic radicals that cause these alterations at the amino acid level, inducing the formation of tyrosine-derived oxidation products, such as di-tyrosine [45]. However, the increase in band intensity indicative of tyrosine is explained by the conversion of phenylalanine into meta- and ortho-tyrosine in the melanin pathway, the latter being a known oxidation product in skin aging [46]. These considerations are supported by a decrease in intensities of deconvoluted band areas assigned to phenylalanine in the spectral region between 590 and 470 cm^−1^ (Figure 6), which will be discussed at a later point.

A significant increase (13.78 to 35.69%) in areas assigned to C-S vibration (710–650 cm^−1^), attributed to methionine or cysteine, is observed for P1_H9_ after exposure to 50 ppm SO_2_ (Figure S17).

This behavior is also reflected in the averaged band areas and hypothesized to represent methionine sulfoxide formation, a common oxidation product in the skin [46]. These findings are accompanied by the decrease of bands assigned to glycine at approx. 700 cm^−1^, changing from 23.78 to 15.21%, and glycine/hydroxyproline at approx. 690 cm^−1^, changing from 16.42 to 7.41%. Currently, there is no hypothesis for this reduction. However, in the statistical analysis, samples aged with 80%RH exhibit stronger differentiation from the rest based on PC2 (Figure 5c). The corresponding loading plot clearly demonstrates that spectral changes observed for glycine, hydroxyproline and tyrosine contribute to the separation along PC1. Furthermore, the noticeable clustering of samples exposed to extreme humid conditions indicates that the variable v(C-S) is highly relevant (Figure 5d).

The exemplary band deconvolution of P5_F7_, as shown in Figure 6, further supports the previously mentioned changes in phenylalanine due to additional aging-induced transitions. The area of the corresponding band component increases (1.33 to 9.21%) after exposure to SO_2_ in combination with 80%RH. Considering that phenylalanine is known to be affected by acids [47], this increase in the band area was expected. It suggests that the oxidizing nature of SO_2_ induces the formation of oxidation products, such as di-tryptophan and its multiple stereoisomers [45].

As depicted in Figure 6, the spectral region between 590 and 470 cm^−1^ primarily captures the stretching motions of disulfide bridges, which play a crucial role in protein folding and the organization of fibrillar, such as connecting α1- and α2-chains in collagen type I (COL1α1 and COL1α2) [48]. Through detailed deconvolution, it becomes possible to analyze sub-bands assigned to three types of cystine rotamers in comparison to the native state: gauche-gauche-gauche (ggg), gauche-gauche-trans (ggt) and trans-gauche-trans (tgt) rotamers, respectively. It is evident that the area of band components corresponding to the most stable ggg conformation undergoes a slight reduction during aging, which is further supported by the obtained averaged band areas. Particularly, the band at 510 cm^−1^ originating from disulfide bonds in the native protein exhibits a significant decrease in the band area (7.21 to 3.20%) [49]. Concurrently, increasing band areas attributed to ggt and tgt conformations are observed, indicating the transition of ggg rotamers into energetically less stable conformations, thereby weakening protein folding before the amide bonds are broken. This transformation is also shown in the statistical analysis, which clearly separates aged samples from their unaged references on PC1 in the score plot, showing a negative correlation with the ggg conformation. When comparing these findings with the results of band deconvolution for light- and humidity-aged samples (Figure S19), it becomes apparent that SO_2_ aging induces the most pronounced transition of cysteine bonds. While humidity-aged samples exhibit a weaker negative correlation between the ggg and tgt conformations in the absence of SO_2_, a positive correlation is observed for light-aged samples.

## 3. Materials and Methods

### 3.1. Parchment Samples

A total of 124 samples of 1 × 1 cm^2^ were cut from six different sheep parchments provided by three manufacturers. Details on the parchment folios are summarized in Table 1 and further completed by Tables S1 and S2. The samples were divided into two batches to study differences for the hair and flesh side.

### 3.2. Artificial Aging with UV/VIS Light, Relative Humidity and SO_2_

Artificial light aging was conducted using a Xenon Arc Simulator lamp in an UVACUBE SOL 2/400F irradiation chamber (Dr. Hönle, Gilching, Germany). The lamp emitted radiation between 295 and 3000 nm. Prior to the aging experiments, the radiation intensity in the UV/VIS range was measured using a UV-Meter Basic (Dr. Hönle, Germany). The measured value of approximately 155 W/m^2^ corresponds to natural aging caused by solar radiation under typical outdoor conditions in Central Europe [29,50]. Light aging experiments were carried out for a total exposure time of 750 h per sample. The experiments were paused and monitored at intervals of 12, 80, 250 and 390 h, as well as after the full exposure time. Temperature inside the chamber was manually measured using a thermometer immediately after each exposure step, and it was maintained at 54 ± 4 °C throughout all experiments.

Weathering experiments involving RH and SO_2_ were performed using a setup that combined a system creating the desired gas atmosphere [51] with a weathering chamber (Bel-ArtTMSP SciencewareTM) made of a co-polyester glass (Purastar^®^, Ha Noi City, Vietnam) to expose the samples. The gas concentration was achieved by humidifying synthetic air 5.0 (Messer, Gumpoldskirchen, Austria) with double-distilled water for RH-aging. For SO_2_ aging, SO_2_ (Messer, Austria) was added to the chamber to achieve a concentration of 50 ppm, which was monitored daily using a SO_2_-specific sensor (Aeroqual Limited, Auckland, New Zealand). In all weathering experiments, a gas flow rate of 100 L/h was maintained to continuously flush the chamber. Three different levels of RH (30, 50 and 80%RH) were applied for one week each.

### 3.3. Colorimetric Analysis by UV/VIS Spectroscopy

Color changes between unaged and aged samples were assessed using a SPM50 Gretag–Macbeth spectrophotometer (XRite, Regensdorf, Switzerland) through colorimetric measurements. Reflectance spectra were acquired with a D65 light source with a 10° standard observer (45°/0° geometry) within the spectral range of 380–730 nm. The instrument was calibrated with an internal white reference. Furthermore, 3 × 3 spots with spot diameters of 1 mm each were analyzed per 1 × 1 cm^2^-sample. Colorimetric values, including the variances of the single color coordinates (Δa* and Δb*), the brightness (ΔL*) and the color variation (ΔE*), were calculated according to the Commission Internationale de l’Èclairage 1976 (CIE 1976) [52].

### 3.4. µ-FTIR Spectroscopy in ATR Mode (µ-ATR/FTIR)

µ-FTIR measurements were performed in ATR mode using a LUMOS FTIR microscope (Bruker Optics, Ettlingen, Germany) equipped with a liquid nitrogen-cooled photoconductive mercury cadmium telluride (PC-MCT) detector, covering an area of 100 × 100 µm^2^. A motorized germanium crystal with a tip diameter of 100 µm and a penetration depth of approximately 0.65 µm, regulated by an internal pressure control, was employed as the ATR probe. Spectra were recorded within the spectral range of 4000–370 cm^−1^ by accumulating 64 scans at a resolution of 4 cm^−1^ A total of 5 × 5 spots were analyzed within an area of 1.0 × 1.5 mm^2^. The resulting IR spectra were averaged, baseline-corrected and vector-normalized. Measurements and data processing were carried out with the software OPUS^®^ (version 8.0, Bruker Optics, Ettlingen, Germany).

### 3.5. µ-Raman Spectroscopy

µ-Raman measurements were performed with a portable Raman microscope, specifically the ProRaman-L-Dual-G (Enwave Optronics, Irvine, CA, USA). The measurements were conducted in a dark room The instrument was equipped with a laser diode emitting light at a wavelength of 785 nm with a linewidth of 2 cm^−1^. A fiber optic probe with a standard working distance of 7.5 mm, coupled with a Rayleigh filter, was used. The Raman signal was detected with a CCD array detector cooled to −60 °C. The integrated microscope featured a 1.3 Megapixel camera with in-line LED illumination. For each sample, five measuring points spaced 2 mm apart were analyzed. The area of interest was visualized using a Leica 50× LWD (long working distance) objective, resulting in an approximate diameter of 3 µm. Spectra were acquired in the spectral range of 3300–100 cm^−1^, using a laser power of approximately 36.9 mW. Each spectrum consists of 10 scans, with an acquisition time of 15 s per spectrum. Subsequently, the obtained Raman spectra were averaged, baseline-corrected and vector-normalized using the software OPUS^®^ (version 7.0, Bruker Optics, Ettlingen, Germany).

### 3.6. Band Deconvolution

The µ-ATR-FTIR and µ-Raman spectra were analyzed using the OriginPro^®^ software (version 2016G, Northampton, MA, USA). To enhance the quality of the spectra, a smoothing technique based on the Savitzky–Golay method was applied. A third order polynomial function and 9 smoothing points were used for this purpose. The spectra contained various absorption bands related to amide and other protein-related vibration, as well as overlapping bands associated with lipids and H-bonds in different regions of interest (Figure S1). To deconvolute these overlapping bands and accurately determine the maxima of the individual bands, curve fitting techniques were employed. The second derivatives of the spectra were utilized to identify the precise location of the band maxima. To reconstruct the enveloping band shape, a Gaussian function was employed as the band shape model. The reconstructed band shape was obtained as a summation of the assigned bands. Detailed information regarding the band assignments can be found in Tables S3 and S4.

### 3.7. Multivariate Data Analysis

The band areas corresponding to the same secondary structure or spectroscopic feature within a single band envelope, such as the α-helix in the amide I band, were combined by summation. This was done before conducting subsequent chemometric analysis. The resulting percentage values were organized in columns based on the band components and in rows based on the samples. They were then exported as a CSV file for further usage.

To examine the differences in the significance of spectral features for parchment degradation under different conditions, the data were subjected to dimensionality reduction using Factor Analysis of Mixed Data (FAMD) as described in the Supplementary Information. The FAMD was performed using the “FAMD” function available in the FactoMiner package in R (version 4.1.2.) [53]. The resulting data were extracted and visualized with the Factorextra [54] package (version 1.0.7.). For each spectral region, the summed percentage band areas of the fitted band components were used as quantitative variables in the analysis. Additionally, qualitative input variables such as the exposed parchment side or the aging state (unaged/aged) were included to provide further information.

## 4. Conclusions

This study presents a comprehensive investigation of parchment degradation, considering not only the structural integrity of collagen but also the results of UV/VIS, µ-ATR/FTIR and µ-Raman measurements. Parchment samples were exposed to light for up to 750 h and subjected to humidity levels of 30, 50 and 80%RH with and without 50 ppm SO_2_. These three degrading agents are recognized as significant factors that require careful regulation and continuous monitoring. The levels of humidity selected in this study aimed to cover appropriate storage conditions (50%RH) as well as extremely dry (30%RH) and humid (80%RH) environments to simulate improper handling situations. Sulfur dioxide was chosen as a representative pollutant due to its historical relevance in contributing to indoor air pollution in ancient times. Despite the significant reduction of indoor and outdoor sulfur dioxide concentrations in recent decades, it played a major role in causing air pollution until the introduction of chimneys in the 12th century [55]. The exposure of parchment to humidity, with and without sulfur dioxide, allowed for investigating the impact of moisture alone and its synergistic interaction with the pollutant.

The combination of complementing spectroscopic methods, supported by multivariate analysis, provided a detailed understanding of the development of degradation-induced modifications in the supramolecular organization of parchment. Shifts in the maximum absorption of the amide I and amide II band, towards higher and lower wavenumbers, respectively, were observed as strong indicators of significant molecular changes of collagen induced by light aging. However, these shifts were not observed after exposure alone or moisture with SO_2_, although changes in the secondary protein structures were observed. Detailed band deconvolution analysis was crucial in revealing molecular changes induced by humidity and SO_2_, as well as the gradual loss of proteinaceous triple-helical structures and the native state of parchment. Advanced statistical analysis, employing factor analysis of mixed data, enabled the identification of spectral changes induced by the individual degrading agents using the fingerprint region of Raman spectra. Specifically, it was found that light and enhanced humidity led to a comparable reduction in protein backbone stability, accompanied by increased crosslink formation due to humidity. Oxidation processes affecting tyrosine, tryptophan or methionine were observed after treatment with light and sulfur dioxide, leading to subsequent cleavage of the protein structure. Additionally, the conformation of disulfide bridges transitioned towards less stable rotamers, resulting in a disrupted fibrillar organization of collagen as a consequence of sulfur dioxide aging. Infrared spectra in the C-H region showed specific markers for light-induced aging effects on lipids in sheep parchment. UV/VIS spectroscopy also revealed differences depending on the aging type: exposure to light and humidity caused darkening of the samples, while sulfur dioxide exposure led to increased brightness associated with progressing brittleness of the sample surfaces. These differences in brightness were further reflected in the color coordinates, showing red/yellow hues for light-aged samples and blue/green hues after exposure to sulfur dioxide.

Previous studies primarily investigated the effects of humidity and pollutants in combination with elevated temperatures. However, the present results demonstrate that even short-term exposures without elevated temperatures can induce degradation. Based on these findings, further long-term aging experiments are planned to investigate the impact of humidity and sulfur dioxide on the material. Additionally, modern atmospheric pollutants such as NO_x_ and O_3_, the latter being still significant as an indoor air pollutant in museums, will be further examined. This study highlights that the established approach of using IR and Raman band shifts to assess environmentally induced aging effects in parchment can be complemented by in-depth statistical analysis of the absorption bands. This approach allows for the identification of molecular changes and assists in early damage assessment. Therefore, the identified spectral features provide valuable additional information to the amide I and II band transitions, particularly when the amide bands are overlapped by traces of the manufacturing process.

## Figures and Tables

**Table 1:** Summary of details of used parchment folios, including notation, manufacturer and known facts regarding preparation ^1^.

Folio	Origin	Age [Years]	Preparation Steps
P1	J. Vnouček	>1	Chalk applied on hair side (in dried state)
P2	J. Vnouček	<1	Chalk applied on hair side (before drying)
P3	J. Vnouček	<1	Chalk applied on hair side
P4	J. Vnouček	<1	Chalk applied on hair side
P5	ARCH Lab	-	-
P6	A. Glaser	-	-

^1^ Exposure of hair and flesh side are further indicated by indices “F” and “H”, e.g., P1_H_ and P1_F_.

## Data Availability

Data are available from the corresponding authors.

## Supplementary Materials

The following supporting information can be downloaded in one file at: https://www.mdpi.com/article/10.3390/molecules28124584/s1, Table S1: Summary of details on parchment folios P1–P4, Table S2: Overview of all samples and their respective aging conditions, Figure S1: Overview of regions of interest (ROI) in (a) ATR/FTIR and (b) Raman spectra analyzed by band fitting. (c) Detail of the lower fingerprint region, Table S3: Band assignment for deconvoluted regions of interest in ATR/FTIR spectra (Figure S1), Table S4: Band assignment for deconvoluted regions of interest in Raman spectra (Figure S1), Figure S2: Representation of the color variation ΔE* after exposure of the samples’ flesh sides to (a) light, (b) humidity, and (c) SO_2_ and the hair sides to (d) light, (e) humidity, and (f) SO_2_. Vertical dashed line marks a threshold of 1 and the perceptibility by the human eye. Figure S3: Results of colorimetric measurements after exposure of flesh and hair sides of P1-P6 to light. Changes of (a) and (d) color variation ΔE*, (b) and (e) brightness ΔL* and (c) and (f) single color coordinates for a second batch of light-aged samples. Figure S4: Results of colorimetric measurements after exposure of the hair sides of P1-P6 to light, humidity and SO_2_. Changes in brightness to (a) light, (b) humidity and (c) SO_2_. Changes of the single color coordinates a* and b* after exposure to (d) light, (e) humidity and (f) SO_2_. Figure S5: Changes in µ-ATR/FTIR spectra between 3600–2800 cm^−1^ of light- and humidity-exposed parchment. Exposure to light results in a decrease of band intensities of the (a) CH-stretching bands (ν_as_(CH_2_) and ν_s_(CH_2_)) and (b) the band envelope centered at 3300 cm^−1^. (c) Increase of band intensities (centered at 3300 cm^−1^) as a result of increasing humidity levels. Figure S6: µ-ATR/FTIR spectra between 3000–2800 cm^−1^ of light-exposed hair side of parchment. (a) Band areas of ν(CH), ν_as_(CH_2_) and ν_s_(CH_2_), ν_as_(CH_3_) and ν_s_(CH_3_) over 750 h of exposure as percentage from the band envelope area (--- 250 h turning point). (b) S-shaped functions observed for intensities of ν_as_(CH_2_) at approx. 2920 cm^−1^ and ν_s_(CH_2_) at approx. 2850 cm^−1^. (c) Blue-shift with progressing exposure time of ν_as_(CH_2_) and ν_s_(CH_2_) bands (in the band envelope). (b–d) represent averaged values for the hair side of all parchment samples exposed. Figure S7: µ-ATR/FTIR spectra between 3600–3000 cm^−1^ of (a) light- and (b) humidity-exposed flesh side of parchment. Band areas of amide A, B and H-bound water-related bands after exposure to (a) light and (b) humidity. Represented averaged values for the flesh side of all parchment samples exposed to (a) light and (b) humidity. Figure S8: µ-ATR/FTIR spectra of parchment P1. (a) Comparison between hair and flesh side. (b) Comparison of hair side with calcite and calcium stearate reference spectra. Figure S9: µ-ATR/FTIR spectra between 1720–1580 cm^−1^ of light-, humidity- and SO_2_- exposed hair side of parchment. Averaged band areas for the amide I band components after (a) light, (b) humidity and (c) SO_2_-expsure as percentage from the band envelope. Figure S10: µ-ATR/FTIR spectra between 1720–1580 cm^−1^ (amide I) and 1590–1480 cm^−1^ (amide II) of light-exposed flesh side of parchment. (a) Exemplary band deconvolution of the amide I band envelope of unaged and 750 h-aged sample P1_F1_. (b) Band areas for amide I band components as percentage of the band envelope. Separation of unaged and aged samples observed in (c) score plots and contributing variables in (d) loading plots. (e) Exemplary band deconvolution of the amide II band envelope of unaged and 750 h-aged sample P1_F1_. (f) Band areas for amide II band components as percentage of the band envelope. Separation of unaged and aged samples observed in (g) score plots and contributing variables in (h) loading plots. Figure S11: µ-Raman spectra between 1425–1150 cm^−1^ of light- and humidity-exposed hair side of parchment. (a) Exemplary band deconvolution of amide III band envelope of unaged and 750 h-aged sample P5_H1_. Band areas for amide III band components after exposure to (b) light and (c) 30%RH/50%RH/80%RH as percentage of the band envelope. Separation of unaged and aged samples observed in score plots and contributing variables in the loading plots (d-e) after light- and (f-g) humidity-exposure. Figure S12: µ-Raman spectra between 1425–1150 cm^−1^ of SO_2_-exposed hair side of parchment. (a) Band areas for amide III band components after exposure to 50 ppm SO_2_ and 30%RH/50%RH/80%RH as percentage of the band envelope. Separation of unaged and aged samples observed in (b) the score plot and contributing variables (c) in the loading plots. Figure S13: µ-Raman spectra between 1425–1150 cm^−1^ after exposure to light, humidity and SO_2_ of flesh side of parchment. Band areas for amide III band components after exposure to (a) light, 30%RH/50%RH/80%RH (b) without and (c) with 50 ppm SO_2_ as percentage of the band envelope. Figure S14: µ-Raman spectra between 1000–800 cm^−1^ of SO_2_-exposed hair side of parchment. (a) Band areas of the respective band components after exposure to 30%RH/50%RH/80%RH and 50 ppm SO_2_ as percentage from the band envelope area. Separation of unaged and aged samples observed in (b) score plots and contributing variables in (c) the loading plots. Figure S15: µ-Raman spectra between 1000–800 cm^−1^ of light-, humidity- and SO_2_-exposed flesh side of parchment. Band areas of the respective band components after exposure to (a) light, 30%RH/50%RH/80%RH (b) without and (c) with 50 ppm SO_2_ as percentage from the band envelope area. Figure S16: µ-Raman spectra between 800–720 cm^−1^ of light, humidity and SO_2_-exposed of parchment. Band areas of the respective band components after exposure to (a) light, 30%RH/50%RH/80%RH (b) without and (c) with 50 ppm SO_2_ as percentage from the band envelope area of flesh sides. (d–f) of exposed hair sides. Figure S17: µ-Raman spectra between 710–650 cm^−1^ of light- and humidity-exposed hair side of parchment. (a) Band areas of the respective band components after light-exposure over 750 h and (d) RH-exposure to 30%RH/50%RH/80%RH as percentage from the band envelope area. Separation of unaged and aged samples observed in score plots and contributing variables in the loading plots (b,c) after light-exposure and (e,f) after RH-aging. Figure S18: µ-Raman spectra between 710–650 cm^−1^ of light-, humidity- and SO_2_-exposed flesh side of parchment. Band areas of the respective band components after exposure to (a) light, 30%RH/50%RH/80%RH (b) without and (c) with 50 ppm SO_2_ as percentage from the band envelope area. Figure S19: µ-Raman spectra between 590–470 cm^−1^ of light- and humidity-exposed hair side of parchment. (a) Band areas of the respective band components after light-exposure over 750 h and (d) RH-exposure to 30%RH/50%RH/80%RH as percentage from the band envelope area. Separation of unaged and aged samples observed in score plots and contributing variables in the loading plots after (b,c) light-exposure and (e,f) humidity-aging. Figure S20: µ-Raman spectra between 590–470 cm^−1^ of light-, humidity-, and SO_2_-exposed parchment. Band areas of the respective band components after exposure from the band envelope area of flesh sides. (d,f) if exposed hair sides. References [56,57,58,59,60,61,62,63,64,65,66,67,68,69,70,71,72,73,74,75,76,77,78,79,80,81,82,83,84,85,86,87,88,89,90,91,92,93,94,95,96,97,98,99,100,101,102,103,104,105,106,107,108,109] are cited in the supplementary materials.

## References

[1] Goggin J.M. (1959). A History of Technology, Volume I: From Early Times to Fall of Ancient Empires. Am. Antiq..

[2] Maor Y., Shor P., Aizenshtat Z. (2021). Parchment Browning–part II: The Dead Sea Scrolls. Polym. Degrad. Stab..

[3] Fuchs R. (2004). The history and biology of parchment. Karger Gaz..

[4] Thompson D.V. (1935). Medieval Parchment-Making.

[5] Kadler K.E., Baldock C., Bella J., Boot-Handford R.P. (2007). Collagens at a glance. J. Cell Sci..

[6] Gordon M.K., Hahn R.A. (2010). Collagens. Cell Tissue Res..

[7] Shoulders M.D., Raines R.T. (2009). Collagen structure and stability. Annu. Rev. Biochem..

[8] Stinson R.H., Sweeny P.R. (1980). Skin collagen has an unusual d-spacing. Biochim. Et Biophys. Acta (BBA)-Protein Struct..

[9] Petruska J.A., Hodge A.J. (1964). A subunit model for the tropocollagen macromolecule. Proc. Natl. Acad. Sci. USA.

[10] Bozec L., van der Heijden G., Horton M. (2007). Collagen fibrils: Nanoscale ropes. Biophys. J..

[11] Kennedy C., Wess T. (2003). The Structure of Collagen within Parchment–A Review. Restaur.-Int. J. Preserv. Libr. Arch. Mater.-Restaur..

[12] Guareschi I. (1905). Della Pergamena, con Osservazioni ed Esperienze sul Ricupero e sul Restauro di Codici Danneggiati Negli Incendi e Notizie Storiche.

[13] Larsen R., European Commission, Directorate-General for Research and Innovation (2007). Improved Damage Assessment of Parchment–IDAP: Assessment, Data Collection and Sharing of Knowledge.

[14] Larsen R. (2002). Microanalysis of Parchment.

[15] Badea E., Usacheva T., Della Gatta G. (2015). The use of differential scanning calorimetry to characterise collagen deterioration in parchment. Ross. Khimicheskij Zhurnal (Zhurnal Ross. Khimicheskogo Obs. Im. D.I. Mendeleeva).

[16] Badea E., Della Gatta G., Usacheva T. (2012). Effects of temperature and relative humidity on fibrillar collagen in parchment: A micro differential scanning calorimetry (micro DSC) study. Polym. Degrad. Stab..

[17] Melniciuc-Puica N., Dorohoi D.O., Melnig V. (2008). Evaluation of parchment chemical degradation. Optoelectron. Adv. Mater.–Rapid Commun..

[18] Riccardi A., Mercuri F., Paoloni S., Zammit U., Marinelli M., Scudieri F. (2010). Parchment ageing study: New methods based on thermal transport and shrinkage analysis. E-Preserv. Sci..

[19] Manfredi M., Bearman G., France F., Shor P., Marengo E. (2015). Quantiative Multispectral Imaging for the Detection of Parchment Ageing Caused by Light: A Comparison with ATR-FTIR, CG-MS and TGA Analyses. Int. J. Conserv. Sci..

[20] Možir A., Strlič M., Trafela T., Cigić I.K., Kolar J., Deselnicu V., de Bruin G. (2011). On oxidative degradation of parchment and its non-destructive characterisation and dating. Appl. Phys. A.

[21] Strlič M., Cigić I.K., Rabin I., Kolar J., Pihlar B., Cassar M. (2009). Autoxidation of lipids in parchment. Polym. Degrad. Stab..

[22] Badea E., Carşote C., Vetter W., Petroviciu I., Miu L., Schreiner M., della Gatta G. How parchment responds to temperature and relative humidity: A combined micro DSC, MHT, SEM and FTIR study. Proceedings of the ICAMS 2012.

[23] Edwards H.G., Perez F.R. (2004). Application of Fourier transform Raman spectroscopy to the characterization of parchment and vellum. II—Effect of biodeterioration and chemical deterioration on spectral interpretation. J. Raman Spectrosc..

[24] Maor Y., Shor P., Aizenshtat Z. (2020). Parchment browning and the Dead Sea Scrolls–Part I: Artificial aging. Polym. Degrad. Stab..

[25] Della Gatta G., Badea E., Ceccarelli R., Usacheva T., Maši A., Coluccia S. (2005). Assessment of damage in old parchments by DSC and SEM. J. Therm. Anal. Calorim..

[26] Kennedy C.J., Wess T.J. (2006). The use of X-ray scattering to analyse parchment structure and degradation. Physical Techniques in the Study of Art, Archaeology and Cultural Heritage.

[27] De Groot J., Odlyha M., Bozec L., Horton M., Masic A., Coluccia S. Damage assessment of parchment by micro-thermal analysis and scanning electron microscopy. Proceedings of the Preprints of the ICOM-CC 14th Triennial Meeting.

[28] Axelsson K.M., Larsen R., Sommer D.V., Melin R. (2016). Degradation of collagen in parchment under the influence of heat-induced oxidation: Preliminary study of changes at macroscopic, microscopic, and molecular levels. Stud. Conserv..

[29] Cappa F., Paganoni I., Carsote C., Badea E., Schreiner M. (2020). Studies on the effects of mixed light-thermal ageing on parchment by vibrational spectroscopy and micro hot table method. Herit. Sci..

[30] Boyatzis S.C., Velivasaki G., Malea E. (2016). A study of the deterioration of aged parchment marked with laboratory iron gall inks using FTIR-ATR spectroscopy and micro hot table. Herit. Sci..

[31] Chadefaux C., Le Hô A.-S., Bellot-Gurlet L., Reiche I. (2009). Curve-fitting Micro-ATR-FTIR studies of the amide I and II bands of type I collagen in archaeological bone materials. E-Preserv. Sci..

[32] Kern M., Pataki-Hundt A., Wouters J., Kirby D. (2018). Accelerated Ageing of Parchment: Investigation of a Photo Catalysed, Low-Heat Approach. Restaurator. Int. J. Preserv. Libr. Arch. Mater..

[33] Bretzendorfer C., Pataki-Hundt A. (2022). Novel Approaches for Opaque Reconstituted Parchment. Restaurator. Int. J. Preserv. Libr. Arch. Mater..

[34] Cappa F., Paganoni I., Carsote C., Schreiner M., Badea E. (2020). Studies on the effect of dry-heat ageing on parchment deterioration by vibrational spectroscopy and micro hot table method. Polym. Degrad. Stab..

[35] Simonot L., Elias M. (2003). Color change due to surface state modification. Color Res. Appl..

[36] Pagnin L., Calvini R., Wiesinger R., Weber J., Schreiner M. (2020). Photodegradation kinetics of alkyd paints: The influence of varying amounts of inorganic pigments on the stability of the synthetic binder. Front. Mater..

[37] Olsztyńska-Janus S., Pietruszka A., Kiełbowicz Z., Czarnecki M.A. (2018). ATR-IR study of skin components: Lipids, proteins and water. Part I: Temperature effect. Spectrochim. Acta Part A Mol. Biomol. Spectrosc..

[38] Boncheva M., Damien F., Normand V. (2008). Molecular organization of the lipid matrix in intact Stratum corneum using ATR-FTIR spectroscopy. Biochim. Et Biophys. Acta (BBA)-Biomembr..

[39] Doherty S., Alexander M.M., Vnouček J., Newton J., Collins M.J. (2021). Measuring the impact of parchment production on skin collagen stable isotope (δ13C and δ15N) values. STAR Sci. Technol. Archaeol. Res..

[40] Možir A., Cigić I.K., Marinšek M., Strlič M. (2014). Material properties of historic parchment: A reference collection survey. Stud. Conserv..

[41] Fourneau M., Canon C., Van Vlaender D., Collins M.J., Fiddyment S., Poumay Y., Deparis O. (2020). Histological study of sheep skin transformation during the recreation of historical parchment manufacture. Herit. Sci..

[42] Rabotyagova O.S., Cebe P., Kaplan D.L. (2008). Collagen Structural Hierarchy and Susceptibility to Degradation by Ultraviolet Radiation. Mater. Sci. Eng. C Mater. Biol. Appl..

[43] Ye H., Rahul, Kruger U., Wang T., Shi S., Norfleet J., De S. (2019). Burn-related Collagen Conformational Changes in ex vivo Porcine Skin using Raman Spectroscopy. Sci. Rep..

[44] Crawford-Manning F., Vardaki M.Z., Green E., Meakin J.R., Vergari C., Stone N., Winlove C.P. (2021). Multiphoton imaging and Raman spectroscopy of the bovine vertebral endplate. Analyst.

[45] Hawkins C.L., Davies M.J. (2019). Detection, identification, and quantification of oxidative protein modifications. J. Biol. Chem..

[46] Wells-Knecht M.C., Lyons T.J., McCance D.R., Thorpe S.R., Baynes J.W. (1997). Age-dependent increase in ortho-tyrosine and methionine sulfoxide in human skin collagen is not accelerated in diabetes. Evidence against a generalized increase in oxidative stress in diabetes. J. Clin. Investig..

[47] Engel B., Suppan J., Nürnberger S., Power A.M., Marchetti-Deschmann M. (2020). Revisiting amino acid analyses for bioadhesives including a direct comparison of tick attachment cement (*Dermacentor marginatus*) and barnacle cement (*Lepas anatifera*). Int. J. Adhes. Adhes..

[48] Simon H.J., Van Agthoven M.A., Lam P.Y., Floris F., Chiron L., Delsuc M.-A., Rolando C., Barrow M.P., O’Connor P.B. (2016). Uncoiling collagen: A multidimensional mass spectrometry study. Analyst.

[49] David C., Foley S., Enescu M. (2010). Mechanisms of Disulfide Bridges Reduction in Lysozyme Revealed by Raman Spectroscopy and Molecular Computing. AIP Conf. Proc..

[50] Šúri M., Huld T.A., Dunlop E.D., Ossenbrink H.A. (2007). Potential of solar electricity generation in the European Union member states and candidate countries. Sol. Energy.

[51] Wiesinger R., Schreiner M., Kleber C. (2010). Investigations of the interactions of CO_2_, O_3_ and UV light with silver surfaces by in situ IRRAS/QCM and ex situ TOF-SIMS. Appl. Surf. Sci..

[52] Johnston-Feller R. (2001). Color Science in the Examination of Museum Objects: Nondestructive Procedures.

[53] Lê S., Josse J., Husson F. (2008). FactoMineR: An R Package for Multivariate Analysis. J. Stat. Softw..

[54] Kassambara A., Mundt F. (2021). Factoextra: Extract and Visualize the Results of Multivariate Data Analyses.

[55] Fowler D., Brimblecombe P., Burrows J., Heal M.R., Grennfelt P., Stevenson D.S., Jowett A., Nemitz E., Coyle M., Liu X. (2020). A chronology of global air quality. Philos. Trans. R. Soc. A.

[56] Barique M.A., Tsuchida E., Ohira A., Tashiro K. (2018). Effect of elevated temperatures on the states of water and their correlation with the proton conductivity of Nafion. ACS Omega.

[57] Lucassen G.W., Van Veen G.N., Jansen J.A. (1998). Band analysis of hydrated human skin stratum corneum attenuated total reflectance Fourier transform infrared spectra in vivo. J. Biomed. Opt..

[58] Wu J.G., Xu Y.Z., Sun C.W., Soloway R.D., Xu D.F., Wu Q.G., Sun K.H., Weng S.F., Xu G.X. (2001). Distinguishing malignant from normal oral tissues using FTIR fiber-optic techniques. Biopolym. Orig. Res. Biomol..

[59] Dovbeshko G.I., Gridina N.Y., Kruglova E.B., Pashchuk O.P. (2000). FTIR spectroscopy studies of nucleic acid damage. Talanta.

[60] Schulz H., Baranska M. (2007). Identification and quantification of valuable plant substances by IR and Raman spectroscopy. Vib. Spectrosc..

[61] Smith R., Rehman I. (1994). Fourier transform Raman spectroscopic studies of human bone. J. Mater. Sci. Mater. Med..

[62] Movasaghi Z., Rehman S., ur Rehman D.I. (2008). Fourier transform infrared (FTIR) spectroscopy of biological tissues. Appl. Spectrosc. Rev..

[63] Eckel R., Huo H., Guan H.-W., Hu X., Che X., Huang W.-D. (2001). Characteristic infrared spectroscopic patterns in the protein bands of human breast cancer tissue. Vib. Spectrosc..

[64] Dovbeshko G., Chegel V., Gridina N.Y., Repnytska O., Shirshov Y., Tryndiak V., Todor I., Solyanik G. (2002). Surface enhanced IR absorption of nucleic acids from tumor cells: FTIR reflectance study. Biopolym. Orig. Res. Biomol..

[65] Yang Y., Sulé-Suso J., Sockalingum G.D., Kegelaer G., Manfait M., El Haj A.J. (2005). Study of tumor cell invasion by Fourier transform infrared microspectroscopy. Biopolym. Orig. Res. Biomol..

[66] Paluszkiewicz C., Kwiatek W.M. (2001). Analysis of human cancer prostate tissues using FTIR microspectroscopy and SRIXE techniques. J. Mol. Struct..

[67] Flach C.R., Moore D.J. (2013). Infrared and Raman imaging spectroscopy of ex vivo skin. Int. J. Cosmet. Sci..

[68] Saeed A., Raouf G.A., Nafee S.S., Shaheen S.A., Al-Hadeethi Y. (2015). Effects of very low dose fast neutrons on cell membrane and secondary protein structure in rat erythrocytes. PLoS ONE.

[69] Arrondo J.L.R., Goni F.M. (1998). Infrared studies of protein-induced perturbation of lipids in lipoproteins and membranes. Chem. Phys. Lipids.

[70] Mendelsohn R., Flach C.R., Moore D.J. (2006). Determination of molecular conformation and permeation in skin via IR spectroscopy, microscopy, and imaging. Biochim. Et Biophys. Acta (BBA)-Biomembr..

[71] Coates J. (2000). Interpretation of infrared spectra, a practical approach. Encyclopedia of Analytical Chemistry.

[72] Barth A. (2007). Infrared spectroscopy of proteins. Biochim. Et Biophys. Acta (BBA)-Bioenerg..

[73] Kavanagh G.M., Clark A.H., Ross-Murphy S.B. (2000). Heat-induced gelation of globular proteins: Part 3. Molecular studies on low pH β-lactoglobulin gels. Int. J. Biol. Macromol..

[74] Kreuzer M., Dučić T., Hawlina M., Andjelic S. (2020). Synchrotron-based FTIR microspectroscopy of protein aggregation and lipids peroxidation changes in human cataractous lens epithelial cells. Sci. Rep..

[75] Goormaghtigh E., Ruysschaert J.-M., Raussens V. (2006). Evaluation of the Information Content in Infrared Spectra for Protein Secondary Structure Determination. Biophys. J..

[76] Adochitei A., Drochioiu G. (2011). Rapid Characterization of peptide secondary structure by FT-IR spectroscopy. Rev. Roum. Chim..

[77] Abu Teir M., Ghithan J., Darwish S., Abu-hadid M. (2012). Multi-spectroscopic investigation of the interactions between cholesterol and human serum albumin. J. Appl. Biol. Sci..

[78] Téllez Soto C.A., Medeiros-Neto L.P., dos Santos L., Santos A.B., Ferreira I., Singh P., Canevari R.A., Martin A.A. (2018). Infrared and confocal Raman spectroscopy to differentiate changes in the protein secondary structure in normal and abnormal thyroid tissues. J. Raman Spectrosc..

[79] Azizova L.R., Kulik T.V., Palianytsia B.B., Zemlyakov A.E., Tsikalova V.N., Chirva V.Y. (2014). Investigation of chemical transformations of thiophenylglycoside of muramyl dipeptide on the fumed silica surface using TPD-MS, FTIR spectroscopy and ES IT MS. Nanoscale Res. Lett..

[80] Mirtič A., Grdadolnik J. (2013). The structure of poly-L-lysine in different solvents. Biophys. Chem..

[81] Sadat A., Joye I.J. (2020). Peak fitting applied to fourier transform infrared and raman spectroscopic analysis of proteins. Appl. Sci..

[82] Byler D.M., Farrell Jr H.M., Susi H. (1988). Raman spectroscopic study of casein structure. J. Dairy Sci..

[83] Peters J., Park E., Kalyanaraman R., Luczak A., Ganesh V. (2016). Protein secondary structure determination using drop coat deposition confocal raman spectroscopy. Spectroscopy.

[84] Rivas-Arancibia S., Rodríguez-Martínez E., Badillo-Ramírez I., López-González U., Saniger J.M. (2017). Structural changes of amyloid beta in hippocampus of rats exposed to ozone: A Raman spectroscopy study. Front. Mol. Neurosci..

[85] Devitt G., Rice W., Crisford A., Nandhakumar I., Mudher A., Mahajan S. (2019). Conformational evolution of molecular signatures during amyloidogenic protein aggregation. ACS Chem. Neurosci..

[86] Martinez M.G., Bullock A.J., MacNeil S., Rehman I.U. (2019). Characterisation of structural changes in collagen with Raman spectroscopy. Appl. Spectrosc. Rev..

[87] Voicescu M., Ionescu S., Nistor C.L. (2017). Spectroscopic study of 3-Hydroxyflavone-protein interaction in lipidic bi-layers immobilized on silver nanoparticles. Spectrochim. Acta Part A Mol. Biomol. Spectrosc..

[88] Herrero A.M. (2008). Raman spectroscopy for monitoring protein structure in muscle food systems. Crit. Rev. Food Sci. Nutr..

[89] Cheng W.T., Liu M.T., Liu H.N., Lin S.Y. (2005). Micro-Raman spectroscopy used to identify and grade human skin pilomatrixoma. Microsc. Res. Tech..

[90] Pezzotti G., Boffelli M., Miyamori D., Uemura T., Marunaka Y., Zhu W., Ikegaya H. (2015). Raman spectroscopy of human skin: Looking for a quantitative algorithm to reliably estimate human age. J. Biomed. Opt..

[91] Shetty G., Kendall C., Shepherd N., Stone N., Barr H. (2006). Raman spectroscopy: Elucidation of biochemical changes in carcinogenesis of oesophagus. Br. J. Cancer.

[92] Stone N., Kendall C., Smith J., Crow P., Barr H. (2004). Raman spectroscopy for identification of epithelial cancers. Faraday Discuss..

[93] Unal M., Jung H., Akkus O. (2016). Novel Raman spectroscopic biomarkers indicate that postyield damage denatures bone’s collagen. J. Bone Miner. Res..

[94] Franzen L., Windbergs M. (2015). Applications of Raman spectroscopy in skin research—From skin physiology and diagnosis up to risk assessment and dermal drug delivery. Adv. Drug Deliv. Rev..

[95] Cárcamo J.J., Aliaga A.E., Clavijo E., Garrido C., Gómez-Jeria J.S., Campos-Vallette M.M. (2012). Proline and hydroxyproline deposited on silver nanoparticles. A Raman, SERS and theoretical study. J. Raman Spectrosc..

[96] Wang S.-s., Ye D.-x., Wang B., Xie C. (2020). The expressions of keratins and P63 in primary squamous cell carcinoma of the thyroid gland: An application of raman spectroscopy. OncoTargets Ther..

[97] Penteado S.C.G., Fogazza B.P., Carvalho C.d.S., Arisawa E.A.L., Martins M.A., Martin A.A., Martinho H.d.S. (2008). Diagnosis of degenerative lesions of supraspinatus rotator cuff tendons by Fourier transform-Raman spectroscopy. J. Biomed. Opt..

[98] Tellez Soto C.A., Pereira L., Dos Santos L., Rajasekaran R., Fávero P., Martin A.A. (2016). DFT: B3LYP/3-21G theoretical insights on the confocal Raman experimental observations in skin dermis of healthy young, healthy elderly, and diabetic elderly women. J. Biomed. Opt..

[99] Movasaghi Z., Rehman S., Rehman I.U. (2007). Raman spectroscopy of biological tissues. Appl. Spectrosc. Rev..

[100] Huang Z., McWilliams A., Lui H., McLean D.I., Lam S., Zeng H. (2003). Near-infrared Raman spectroscopy for optical diagnosis of lung cancer. Int. J. Cancer.

[101] Gniadecka M., Wulf H., Nymark Mortensen N., Faurskov Nielsen O., Christensen D.H. (1997). Diagnosis of basal cell carcinoma by Raman spectroscopy. J. Raman Spectrosc..

[102] Aliaga A., Osorio-Román I., Leyton P., Garrido C., Carcamo J., Caniulef C., Celis F., Díaz F. G., Clavijo E., Gómez-Jeria J. (2009). Surface-enhanced Raman scattering study of L-tryptophan. J. Raman Spectrosc. Int. J. Orig. Work All Asp. Raman Spectrosc. Incl. High. Order Process. Also Brillouin Rayleigh Scatt..

[103] Pinheiro A.L.B., Santos N.R.S., Oliveira P.C., Aciole G.T.S., Ramos T.A., Gonzalez T.A., da Silva L.N., Barbosa A.F.S., Silveira L. (2013). The efficacy of the use of IR laser phototherapy associated to biphasic ceramic graft and guided bone regeneration on surgical fractures treated with wire osteosynthesis: A comparative laser fluorescence and Raman spectral study on rabbits. Lasers Med. Sci..

[104] Frank C.J., McCreery R.L., Redd D.C. (1995). Raman spectroscopy of normal and diseased human breast tissues. Anal. Chem..

[105] Frushour B.G., Koenig J.L. (1975). Raman scattering of collagen, gelatin, and elastin. Biopolym. Orig. Res. Biomol..

[106] Rygula A., Majzner K., Marzec K.M., Kaczor A., Pilarczyk M., Baranska M. (2013). Raman spectroscopy of proteins: A review. J. Raman Spectrosc..

[107] Dehring K.A., Smukler A.R., Roessler B.J., Morris M.D. (2006). Correlating changes in collagen secondary structure with aging and defective type II collagen by Raman spectroscopy. Appl. Spectrosc..

[108] Guilbert M., Said G., Happillon T., Untereiner V., Garnotel R., Jeannesson P., Sockalingum G.D. (2013). Probing non-enzymatic glycation of type I collagen: A novel approach using Raman and infrared biophotonic methods. Biochim. Et Biophys. Acta (BBA)-Gen. Subj..

[109] Brandt N.N., Chikishev A.Y., Mankova A.A., Sakodynskaya I.K. (2015). Effect of thermal denaturation, inhibition, and cleavage of disulfide bonds on the low-frequency Raman and FTIR spectra of chymotrypsin and albumin. J. Biomed. Opt..

