# Supplementary material for: Spectral Features Differentiate Aging-Induced Changes in Parchment—A Combined Approach of UV/VIS, µ-ATR/FTIR and µ-Raman Spectroscopy with Multivariate Data Analysis
Source: Molecules. 2023 Jun 6;28(12):4584. doi: 10.3390/molecules28124584 (PMC10302916; doi:10.3390/molecules28124584)
Supplement: Supplementary file 1 [file molecules-28-04584-s001.zip › molecules-2389693-supplementary.pdf]

SUPPLEMENTARY INFORMATION

Spectral Features Differentiate Aging-Induced Changes in Parchment – A Combined Approach of UV/VIS, µ-ATR/FTIR and µ-Raman Spectroscopy with Multivariate Data Analysis

Antonia Malissa [1,2,*] , Federica Cappa [2] , Manfred Schreiner [1,2] and Martina Marchetti-Deschmann [1,*]

1 Institute of Chemical Technologies and Analytics, TU Wien, Getreidemarkt 9, A-1060 Vienna, Austria

2 Institute of Science and Technology in Art, Academy of Fine Arts Vienna, Schillerplatz 3, A-1010 Vienna, Austria

* Correspondence: antonia.malissa@tuwien.ac.at (A.M.); martina.marchetti-deschmann@tuwien.ac.at (M.M.-D.)

Table of Contents

	Details on parchment folios
**Table S1**	Summary of details on parchment folios P1-P4.
**Table S2**	Overview of all samples and their respective aging conditions.
**Figure S1**	Overview of regions of interest (ROI) in (a) ATR/FTIR and (b) Raman spectra analyzed by band fitting. (c) Detail of the lower fingerprint region.
**Table S3**	Band assignment for deconvoluted regions of interest in ATR/FTIR spectra (Figure S1).
**Table S4**	Band assignment for deconvoluted regions of interest in Raman spectra (Figure S1).
**Figure S2**	Representation of the color variation ΔE* after exposure of the samples' flesh sides to (a) light, (b) humidity, and (c) SO_2 and the hair sides to (d) light, (e) humidity, and (f) SO_2. Vertical dashed line marks a threshold of 1 and the perceptibility by the human eye.
**Figure S3**	Results of colorimetric measurements after exposure of flesh and hair sides of P1-P6 to light. Changes of (a) and (d) color variation ΔE*, (b) and € brightness ΔL* and (c) and (f) single color coordinates for a second batch of light-aged samples.
**Figure S4**	Results of colorimetric measurements after exposure of the hair sides of P1-P6 to light, humidity and SO_2. Changes in brightness to (a) light, (b) humidity and (c) SO_2. Changes of the single color coordinates a* and b* after exposure to (d) light, (e) humidity and (f) SO_2.
**Figure S5**	Changes in µ-ATR/FTIR spectra between 3600-2800 cm^{-1} of light- and humidity-exposed parchment. Exposure to light results in a decrease of band intensities of the (a) CH-stretching bands ($\nu_{as}(CH_2)$ and $\nu_s(CH_2)$) and (b) the band envelope centered at 3300 cm^{-1}. (c) Increase of band intensities (centered at 3300 cm^{-1}) as a result of increasing humidity levels.
**Figure S6**	µ-ATR/FTIR spectra between 3000-2800 cm^{-1} of light-exposed hair side of parchment. (a) Band areas of ν(CH), $\nu_{as}(CH_2)$ and $\nu_s(CH_2)$, $\nu_{as}(CH_3)$ and $\nu_s(CH_3)$ over 750 h of exposure as percentage from the band envelope area (--- 250 h turning point). (b) S-shaped functions observed for intensities of $\nu_{as}(CH_2)$ at approx. 2920 cm^{-1} and $\nu_s(CH_2)$ at approx. 2850 cm^{-1}. (c) Blue-shift with progressing exposure time of $\nu_{as}(CH_2)$ and $\nu_s(CH_2)$ bands (in the band envelope). (b)-(d) represent averaged values for the hair side of all parchment samples exposed.
**Figure S7**	µ-ATR/FTIR spectra between 3600-3000 cm^{-1} of (a) light- and (b) humidity-exposed flesh side of parchment. Band areas of amide A, B and H-bound water-related bands after exposure to (a) light and (b) humidity. Represented averaged values for the flesh side of all parchment samples exposed to (a) light and (b) humidity.
**Figure S8**	µ-ATR/FTIR spectra of parchment P1. (a) Comparison between hair and flesh side. (b) Comparison of hair side with calcite and calcium stearate reference spectra.
**Figure S9**	µ-ATR/FTIR spectra between 1720-1580 cm^{-1} of light-, humidity- and SO_2- exposed hair side of parchment. Averaged band areas for the amide I band components after (a) light, (b) humidity and (c) SO_2-expsure as percentage from the band envelope.
**Figure S10**	µ-ATR/FTIR spectra between 1720-1580 cm^{-1} (amide I) and 1590-1480 cm^{-1} (amide II) of light-exposed flesh side of parchment. (a) Exemplary band deconvolution of the amide I band envelope of unaged and 750 h-aged sample $P1_{F1}$. (b) Band areas for amide I band components as percentage of the band envelope. Separation of unaged and aged samples observed in (c) score plots and contributing variables in (d) loading plots. (e) Exemplary band deconvolution of the amide II band envelope of unaged and 750 h-aged sample $P1_{F1}$. (f) Band areas for amide II band components as percentage of the band envelope. Separation of unaged and aged samples observed in (g) score plots and contributing variables in (h) loading plots.
**Figure S11**	µ-Raman spectra between 1425-1150 cm-1 of light- and humidity-exposed hair side of parchment. (a) Exemplary band deconvolution of amide III band envelope of unaged and 750 h-aged sample $P5_{H1}$. Band areas for amide III band components after exposure to (b) light and (c) 30%RH/50%RH/80%RH as percentage of the band envelope. Separation of unaged and aged samples observed in score plots and contributing variables in the loading plots (d-e) after light- and (f-g) humidity-exposure.
**Figure S12**	µ-Raman spectra between 1425-1150 cm^{-1} of SO_2-exposed hair side of parchment. (a) Band areas for amide III band components after exposure to 50 ppm SO_2 and 30%RH/50%RH/80%RH as percentage of the band envelope. Separation of unaged and aged samples observed in (b) the score plot and contributing variables (c) in the loading plots.
**Figure S13**	µ-Raman spectra between 1425-1150 cm^{-1} after exposure to light, humidity and SO_2 of flesh side of parchment. Band areas for amide III band components after exposure to (a) light, 30%RH/50%RH/80%RH (b) without and (c) with 50 ppm SO_2 as percentage of the band envelope.

Figure S14 μ-Raman spectra between 1000-800 cm^{-1} of SO_2-exposed hair side of parchment. (a) Band areas of the respective band components after exposure to 30%RH/50%RH/80%RH and 50 ppm SO_2 as percentage from the band envelope area. Separation of unaged and aged samples observed in (b) score plots and contributing variables in (c) the loading plots.

Figure S15 μ-Raman spectra between 1000-800 cm^{-1} of light-, humidity- and SO_2-exposed flesh side of parchment. Band areas of the respective band components after exposure to (a) light, 30%RH/50%RH//80%RH (b) without and (c) with 50 ppm SO_2 as percentage from the band envelope area.

Figure S16 μ-Raman spectra between 800-720 cm^{-1} of light, humidity and SO_2-exposed of parchment. Band areas of the respective band components after exposure to (a) light, 30%RH/50%RH//80%RH (b) without and (c) with 50 ppm SO_2 as percentage from the band envelope area of flesh sides. (d)-(f) of exposed hair sides.

Figure S17 μ-Raman spectra between 710-650 cm^{-1} of light- and humidity-exposed hair side of parchment. (a) Band areas of the respective band components after light-exposure over 750 h and (d) RH-exposure to 30%RH/50%RH/80%RH as percentage from the band envelope area. Separation of unaged and aged samples observed in score plots and contributing variables in the loading plots (b-c) after light-exposure and (e-f) after RH-aging.

Figure S18 μ-Raman spectra between 710-650 cm^{-1} of light-, humidity- and SO_2-exposed flesh side of parchment. Band areas of the respective band components after exposure to (a) light, 30%RH/50%RH/80%RH (b) without and (c) with 50 ppm SO_2 as percentage from the band envelope area.

Figure S19 μ-Raman spectra between 590-470 cm^{-1} of light- and humidity-exposed hair side of parchment. (a) Band areas of the respective band components after light-exposure over 750 h and (d) RH-exposure to 30%RH/50%RH/80%RH as percentage from the band envelope area. Separation of unaged and aged samples observed in score plots and contributing variables in the loading plots after (b-c) light-exposure and (e-f) humidity-aging.

Figure S20 μ-Raman spectra between 590-470 cm^{-1} of light-, humidity-, and SO_2-exposed parchment. Band areas of the respective band components after exposure from the band envelope area of flesh sides. (d-f) if exposed hair sides.

References

Details on Parchment Samples

Four folios (P1-P4) were obtained from a private parchment maker (J. Vnoucek, Czech Republic), whereas parchment P5 was provided by the *Advanced Research for Cultural Heritage Laboratory* (ARCH Lab) of the National Research & Development Institute for Textiles and Leather (INCDTP-ICPI, Romania). Parchment P6 was purchased from the company A. Glaser (Germany). Details on the manufacturing process were only provided for parchments P1-P4 and are summarized in Table S1.

Table S1. Summary of details on parchment folios P1-P4.

Parchment	Details	General Observations
P1	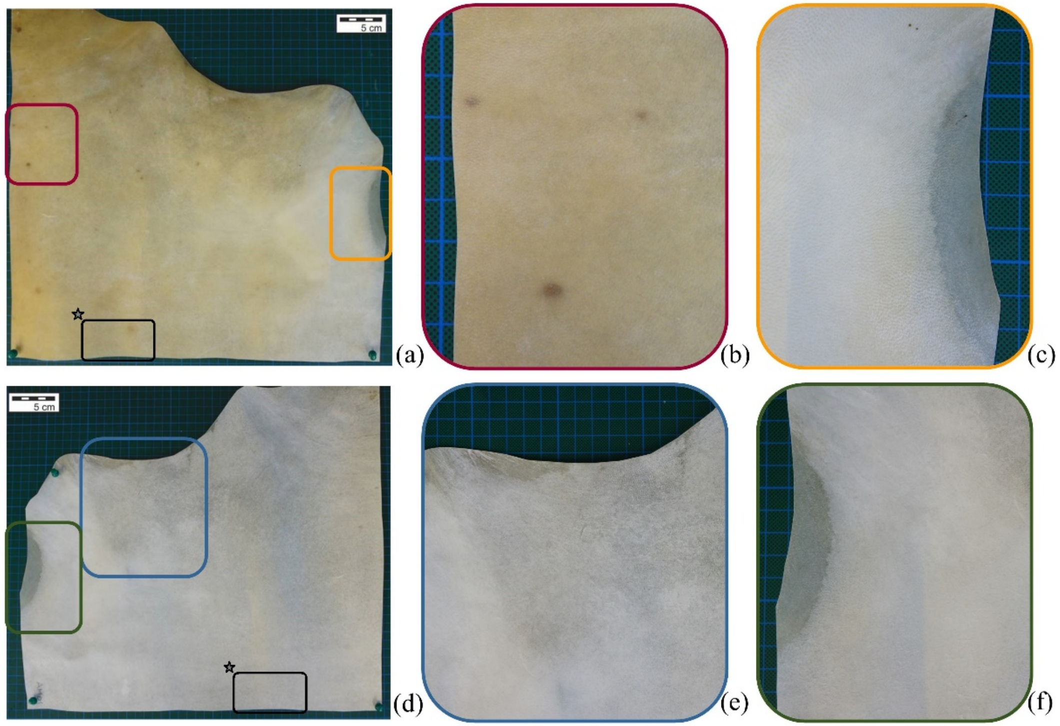	In comparison with folio P2-P4, parchment P1 was obtained from an approx. 1 year old animal, resulting in a higher thickness and higher level of fat content.<br>• Hair (a-c) is darker than the flesh side (d-f) showing a clearly visible follicle pattern.<br>• Transparent areas on the edges (c and d) indicate gelatinization of this part of the sample.<br>• The sampling region is marked in black (*).

Parchment	Details	General Observations
P2	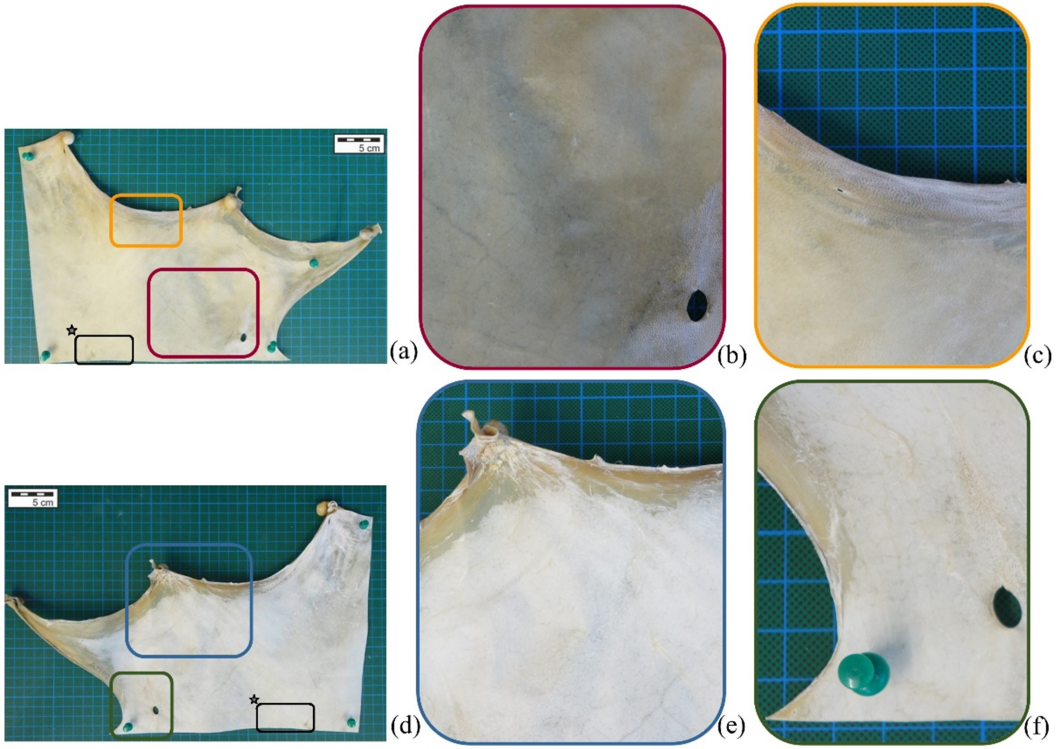	• Hair (a-c) is darker than the flesh side (d-f) of folio P2 show a striking difference in color, with the hair side being darker.<br><br>• In comparison to P1 the follicle pattern is only visible in a few areas (b-c).<br><br>• Furthermore, P2 shows even more significant signs of starting gelatinization on some edges of the folio resulting in brownish transparent, partially hardened areas (d-f).<br><br>• The most distinctive difference between P1 and P2 is the applied surface treatment approach, resulting in a different appearance of the flesh sides. According to the producer, all preparation steps after the assembling of the skin to the wooden frame – including cleaning of the flesh side and the application of chalk – were performed in a wet stage. Therefore, it was dried without further touching the parchment, resulting in a closed and glossy surface (e).<br><br>• The sampling region is marked in black (*).

Parchment	Details	General Observations
P3	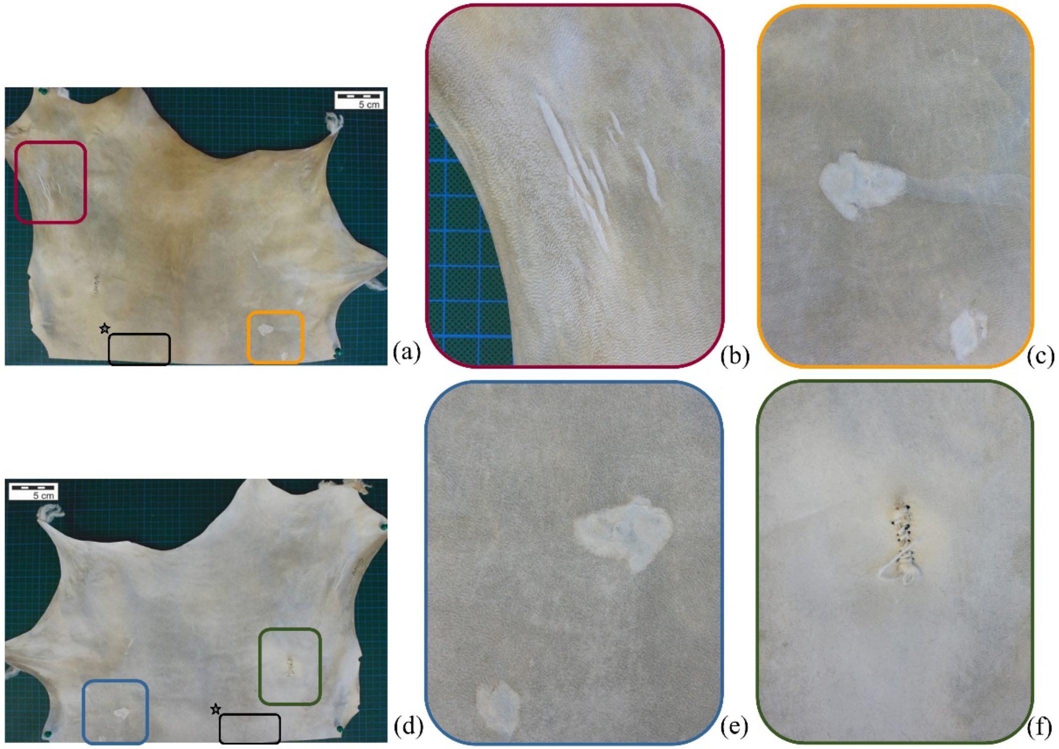	• Hair (a-c) and flesh side (d-f) of folio P3 can be clearly distinguished based on their different color.<br>• A distinct follicle pattern is visible on the hair side (b-c).<br>• According to the manufacturer, the surface of the flesh side was partially treated with a pumice stone to smooth imperfect parts, while the rest of the folio was left without further treatment. Therefore, the surface of this folio partially shows an open, rough structure (comparable with P1) as well as closed, glossy areas comparable with P2.<br>• Several imperfections resulting from the production process are clearly visible (b-d).<br>• The sampling region is marked in black (*).

Parchment	Details	General Observations
P4	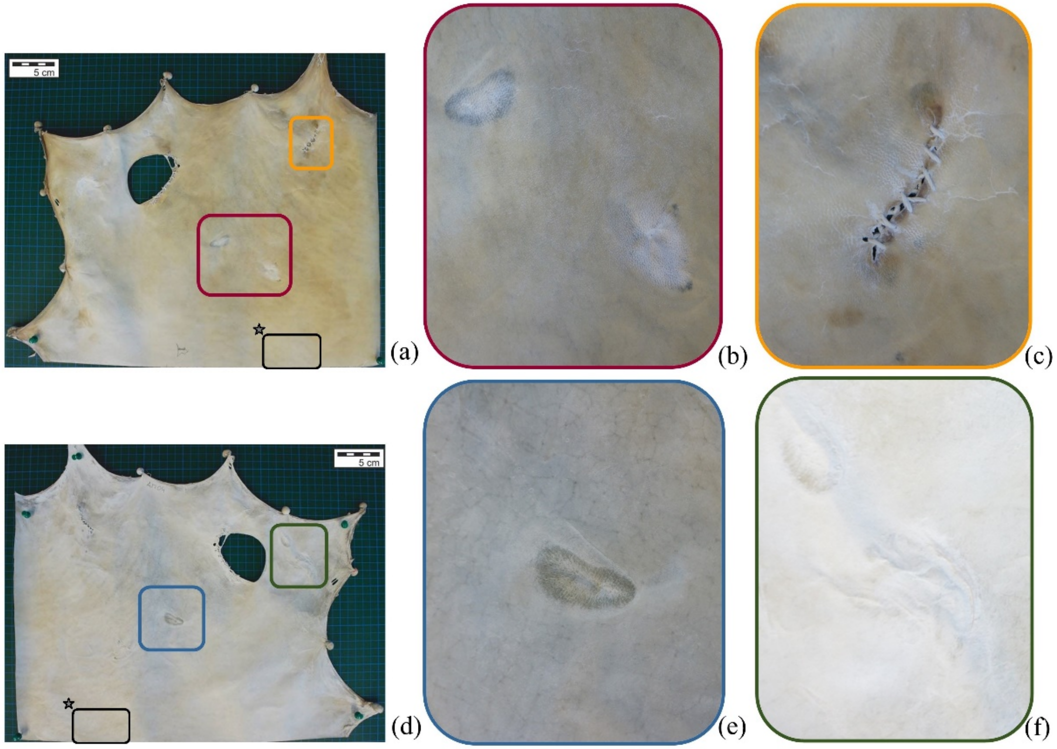	• Also the hair (a-c) side of folio P4 appears darker than the flesh side (d-f).<br>• A distinct follicle pattern is visible on the hair side (b-c).<br>• The surface of the parchment appears smooth and glossy.<br>• A high number of imperfections resulting from the preparation process, e.g. delamination and stitched holes, are evident on both sides (b-c, e-f).<br>• The sampling region is marked in black (*).

Table S2. Overview of all samples and their respective aging conditions.

Sample	Light-aging Description	Conditions	Humidity-aging Description	Conditions	SO_2-aging Description	Conditions
**P1$_{F1}$**	Flesh side, up	155 W/m^2, t_0-$t_{750\,h}$				
**P1$_{F2}$**	Flesh side, up	155 W/m^2, t_0-$t_{750\,h}$				
**P1$_{H1}$**	Hair side, up	155 W/m^2, t_0-$t_{750\,h}$				
**P1$_{H2}$**	Hair side, up	155 W/m^2, t_0-$t_{750\,h}$				
**P2$_{F1}$**	Flesh side, up	155 W/m^2, t_0-$t_{750\,h}$				
**P2$_{F2}$**	Flesh side, up	155 W/m^2, t_0-$t_{750\,h}$				
**P2$_{H1}$**	Hair side, up	155 W/m^2, t_0-$t_{750\,h}$				
**P2$_{H2}$**	Hair side, up	155 W/m^2, t_0-$t_{750\,h}$				
**P3$_{F1}$**	Flesh side, up	155 W/m^2, t_0-$t_{750\,h}$				
**P3$_{F2}$**	Flesh side, up	155 W/m^2, t_0-$t_{750\,h}$				
**P3$_{H1}$**	Hair side, up	155 W/m^2, t_0-$t_{750\,h}$				
**P3$_{H2}$**	Hair side, up	155 W/m^2, t_0-$t_{750\,h}$				
**P4$_{F1}$**	Flesh side, up	155 W/m^2, t_0-$t_{750\,h}$				
**P4$_{F2}$**	Flesh side, up	155 W/m^2, t_0-$t_{750\,h}$				
**P4$_{H1}$**	Hair side, up	155 W/m^2, t_0-$t_{750\,h}$				
**P4$_{H2}$**	Hair side, up	155 W/m^2, t_0-$t_{750\,h}$				
**P5$_{F1}$**	Flesh side, up	155 W/m^2, t_0-$t_{750\,h}$				
**P5$_{H1}$**	Hair side, up	155 W/m^2, t_0-$t_{750\,h}$				
**P6$_{F1}$**	Flesh side, up	155 W/m^2, t_0-$t_{750\,h}$				
**P6$_{F2}$**	Flesh side, up	155 W/m^2, t_0-$t_{750\,h}$				
**P6$_{H1}$**	Hair side, up	155 W/m^2, t_0-$t_{750\,h}$				
**P6$_{H2}$**	Hair side, up	155 W/m^2, t_0-$t_{750\,h}$				
**P1$_{F5}$**			Flesh Side, up	30 %RH, 1 week, RT		
**P1$_{H5}$**			Hair Side, up	30 %RH, 1 week, RT		
**P2$_{F5}$**			Flesh Side, up	30 %RH, 1 week, RT		
**P2$_{H5}$**			Hair Side, up	30 %RH, 1 week, RT		
**P3$_{F5}$**			Flesh Side, up	30 %RH, 1 week, RT		
**P3$_{H5}$**			Hair Side, up	30 %RH, 1 week, RT		
**P4$_{F5}$**			Flesh Side, up	30 %RH, 1 week, RT		
**P4$_{H5}$**			Hair Side, up	30 %RH, 1 week, RT		
**P5$_{F3}$**			Flesh Side, up	30 %RH, 1 week, RT		
**P5$_{H3}$**			Hair Side, up	30 %RH, 1 week, RT		
**P6$_{F3}$**			Flesh Side, up	30 %RH, 1 week, RT		

Sample	Light-aging Description	Conditions	Humidity-aging Description	Conditions	SO_2-aging Description	Conditions
**P6$_{H3}$**			Hair Side, up	30 %RH, 1 week, RT		
**P1$_{F6}$**			Flesh Side, up	50 %RH, 1 week, RT		
**P1$_{H6}$**			Hair Side, up	50 %RH, 1 week, RT		
**P2$_{F6}$**			Flesh Side, up	50 %RH, 1 week, RT		
**P2$_{H6}$**			Hair Side, up	50 %RH, 1 week, RT		
**P3$_{F6}$**			Flesh Side, up	50 %RH, 1 week, RT		
**P3$_{H6}$**			Hair Side, up	50 %RH, 1 week, RT		
**P4$_{F6}$**			Flesh Side, up	50 %RH, 1 week, RT		
**P4$_{H6}$**			Hair Side, up	50 %RH, 1 week, RT		
**P5$_{F4}$**			Flesh Side, up	50 %RH, 1 week, RT		
**P5$_{H4}$**			Hair Side, up	50 %RH, 1 week, RT		
**P6$_{F4}$**			Flesh Side, up	50 %RH, 1 week, RT		
**P6$_{H4}$**			Hair Side, up	50 %RH, 1 week, RT		
**P1$_{F7}$**			Flesh Side, up	80 %RH, 1 week, RT		
**P1$_{H7}$**			Hair Side, up	80 %RH, 1 week, RT		
**P2$_{F7}$**			Flesh Side, up	80 %RH, 1 week, RT		
**P2$_{H7}$**			Hair Side, up	80 %RH, 1 week, RT		
**P3$_{F7}$**			Flesh Side, up	80 %RH, 1 week, RT		
**P3$_{H7}$**			Hair Side, up	80 %RH, 1 week, RT		
**P4$_{F7}$**			Flesh Side, up	80 %RH, 1 week, RT		
**P4$_{H7}$**			Hair Side, up	80 %RH, 1 week, RT		
**P5$_{F5}$**			Flesh Side, up	80 %RH, 1 week, RT		
**P5$_{H5}$**			Hair Side, up	80 %RH, 1 week, RT		
**P6$_{F5}$**			Flesh Side, up	80 %RH, 1 week, RT		
**P6$_{H5}$**			Hair Side, up	80 %RH, 1 week, RT		
**P1$_{F3}$**					Flesh Side, up	50 ppm SO_2, 30 %RH, 1 week, RT
**P1$_{F4}$**					Flesh Side, up	50 ppm SO_2, 30 %RH, 1 week, RT
**P1$_{H3}$**					Hair Side, up	50 ppm SO_2, 30 %RH, 1 week, RT
**P1$_{H4}$**					Hair Side, up	50 ppm SO_2, 30 %RH, 1 week, RT
**P2$_{F3}$**					Flesh Side, up	50 ppm SO_2, 30 %RH, 1 week, RT
**P2$_{F4}$**					Flesh Side, up	50 ppm SO_2, 30 %RH, 1 week, RT
**P2$_{H3}$**					Hair Side, up	50 ppm SO_2, 30 %RH, 1 week, RT
**P2$_{H4}$**					Hair Side, up	50 ppm SO_2, 30 %RH, 1 week, RT
**P3$_{F3}$**					Flesh Side, up	50 ppm SO_2, 30 %RH, 1 week, RT
**P3$_{F4}$**					Flesh Side, up	50 ppm SO_2, 30 %RH, 1 week, RT
**P3$_{H3}$**					Hair Side, up	50 ppm SO_2, 30 %RH, 1 week, RT

Sample	Light-aging Description	Conditions	Humidity-aging Description	Conditions	SO_2-aging Description	Conditions
**P3**$_{H4}$					Hair Side, up	50 ppm SO_2, 30 %RH, 1 week, RT
**P4**$_{F3}$					Flesh Side, up	50 ppm SO_2, 30 %RH, 1 week, RT
**P4**$_{F4}$					Flesh Side, up	50 ppm SO_2, 30 %RH, 1 week, RT
**P4**$_{H3}$					Hair Side, up	50 ppm SO_2, 30 %RH, 1 week, RT
**P4**$_{H3}$					Hair Side, up	50 ppm SO_2, 30 %RH, 1 week, RT
**P5**$_{F2}$					Flesh Side, up	50 ppm SO_2, 30 %RH, 1 week, RT
**P5**$_{H2}$					Hair Side, up	50 ppm SO_2, 30 %RH, 1 week, RT
**P6**$_{F8}$					Flesh Side, up	50 ppm SO_2, 30 %RH, 1 week, RT
**P6**$_{F9}$					Flesh Side, up	50 ppm SO_2, 30 %RH, 1 week, RT
**P6**$_{H8}$					Hair Side, up	50 ppm SO_2, 30 %RH, 1 week, RT
**P6**$_{H9}$					Hair Side, up	50 ppm SO_2, 30 %RH, 1 week, RT
**P1**$_{F8}$					Flesh Side, up	50 ppm SO_2, 50 %RH, 1 week, RT
**P1**$_{H8}$					Hair Side, up	50 ppm SO_2, 50 %RH, 1 week, RT
**P2**$_{F8}$					Flesh Side, up	50 ppm SO_2, 50 %RH, 1 week, RT
**P2**$_{H8}$					Hair Side, up	50 ppm SO_2, 50 %RH, 1 week, RT
**P3**$_{F8}$					Flesh Side, up	50 ppm SO_2, 50 %RH, 1 week, RT
**P3**$_{H8}$					Hair Side, up	50 ppm SO_2, 50 %RH, 1 week, RT
**P4**$_{F8}$					Flesh Side, up	50 ppm SO_2, 50 %RH, 1 week, RT
**P4**$_{H8}$					Hair Side, up	50 ppm SO_2, 50 %RH, 1 week, RT
**P5**$_{F6}$					Flesh Side, up	50 ppm SO_2, 50 %RH, 1 week, RT
**P5**$_{H6}$					Hair Side, up	50 ppm SO_2, 50 %RH, 1 week, RT
**P6**$_{F6}$					Flesh Side, up	50 ppm SO_2, 50 %RH, 1 week, RT
**P6**$_{H6}$					Hair Side, up	50 ppm SO_2, 50 %RH, 1 week, RT
**P1**$_{F9}$					Flesh Side, up	50 ppm SO_2, 80 %RH, 1 week, RT
**P1**$_{H9}$					Hair Side, up	50 ppm SO_2, 80 %RH, 1 week, RT
**P2**$_{F9}$					Flesh Side, up	50 ppm SO_2, 80 %RH, 1 week, RT
**P2**$_{H9}$					Hair Side, up	50 ppm SO_2, 80 %RH, 1 week, RT
**P3**$_{F9}$					Flesh Side, up	50 ppm SO_2, 80 %RH, 1 week, RT
**P3**$_{H9}$					Hair Side, up	50 ppm SO_2, 80 %RH, 1 week, RT
**P4**$_{F9}$					Flesh Side, up	50 ppm SO_2, 80 %RH, 1 week, RT
**P4**$_{H9}$					Hair Side, up	50 ppm SO_2, 80 %RH, 1 week, RT
**P5**$_{F7}$					Flesh Side, up	50 ppm SO_2, 80 %RH, 1 week, RT
**P5**$_{H7}$					Hair Side, up	50 ppm SO_2, 80 %RH, 1 week, RT
**P6**$_{F7}$					Flesh Side, up	50 ppm SO_2, 80 %RH, 1 week, RT
**P6**$_{H7}$					Hair Side, up	50 ppm SO_2, 80 %RH, 1 week, RT

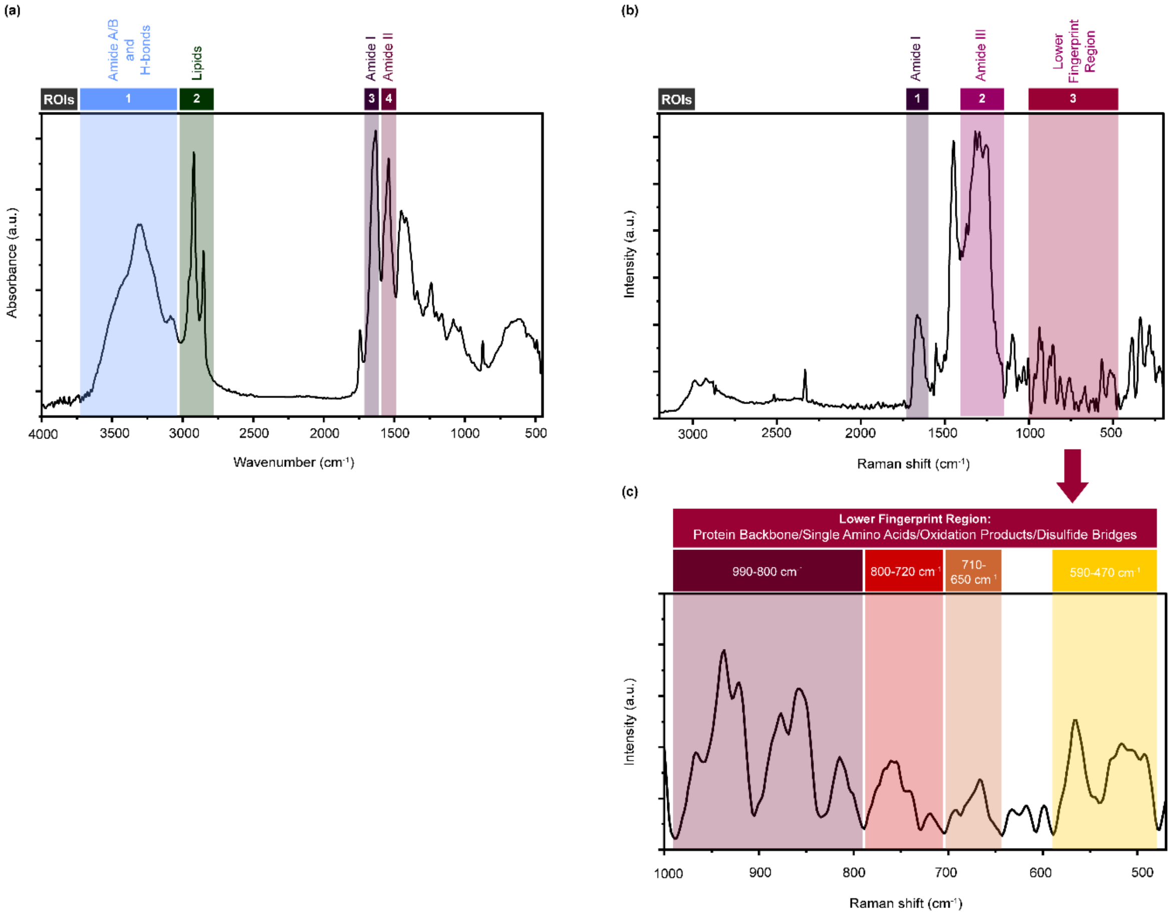

Figure S1. Overview of regions of interest (ROI) in (a) ATR-FTIR and (b) Raman spectra analyzed by band fitting. (c) Detail of the lower fingerprint region.

Table S3. Band assignment for deconvoluted regions of interest in ATR/FTIR spectra (Figure S1).

ROI	Deconvoluted range [cm^{-1}]	Spectral range [cm^{-1}]	Assignment	Reference
1: Amide A/B and H-bonds	3700-3000	*3700-3000*	*ν(OH) of water*	
		3693	ν(OH): non-H-bonded water (dangling)	[1]
		3615	ν_s(OH): free water molecules	[2, 3]
		3611	ν(OH) and ν(NH)	[4]
		3600-3500	*OH-bonds*	
		3599-3570	ν(OH)	[5]
		3561	ν(OH): free	[6]
		3541-3524	ν(OH)	[5]
		3506	ν(OH): free	[7]
		3550-3200	ν_{as} and ν_s attributed to water	[8]
		3500-3470	intermolecular H-bonds	[9]
		3490	ν_{as}(OH): water molecules	[2]
		3450	ν(NH) coupled with H-bond from water	[9]
		3442-3435	ν_{as}(OH)	[5]
		3430	ν(NH) of mainly trans-ordered substructures	[10]
		3420	ν_s(OH): water molecules	[2, 3]
		3419-3410	ν_{as}(OH)	[5]
		3400	ν_s(OH): water molecules	[2]
		3396	ν_{as}(OH)	[5]
		3359-3350	ν(OH), ν(NH), ν(CH)	[11]
		3343-3330	ν_{as}(NH)	[5]
		3325-3295	*amide A*	[12, 13]
		3325-3300	amide A: ν(NH)	[9]
		3300	amide A: ν(NH), hydrogen bonds	[2, 3, 14]
		3217-3180	amide A: ν_{as}(-CH_2=N-)	[9]
		3293	ν(OH): associated	[6]
		3289-3273	ν_s(OH)	[3]
		3280	amide B: ν(NH), hydrogen bonds	[2]

ROI	Deconvoluted range [cm^{-1}]	Spectral range [cm^{-1}]	Assignment	Reference
		3226-3216	ν_s(OH)	[3]
		3200-3194	ν_s(NH)	[5]
		3190	ν(NH) of mainly cis-ordered substructures	[10]
		3182	ν_s(NH)	[5]
		3163	ν_s(NH)	[5]
		3116-3111	CH-ring	[5]
		3078	CH-ring	[5]
		3100-3060	*amide B*	
		3100	amide B: ν(NH), hydrogen bonds	[2, 14]
		3080	amide B: ν(NH)	[9]
		3075	amide B	[5]
		3070	amide II: Fermi-enhanced overtone of 1550 cm^{-1}	[3]
		3060	amide B: ν(NH)	[15]
		3050	amide B	[13]
2: Lipids	3000-2800	2990-2945	$\nu_{as}(CH_3)$	[2, 3, 14-16]
		2945-2905	$\nu_{as}(CH_2)$	[2, 3, 14-17]
		2904-2894	ν(CH)	[18]
		2885-2861	$\nu_s(CH_3)$	[2, 3, 14-16]
		2860-2840	$\nu_s(CH_2)$	[2, 3, 14, 16, 17]
3: Amide I	1700-1580	*1698-1670*	*anti-parallel β-sheets, intermolecular*	
		1698-1680	anti-parallel β-sheets, intermolecular OR β-turn	[2, 19, 20]
		1679-1670	anti-parallel β-sheets, intermolecular	[2, 19, 21]
		1669-1661	β-turn	[2, 19, 20]
		1660-1659	3_{10}-helix	[2]
		1658-1648	α-helix	[2, 9, 19, 21, 22]
		1647-1641	unordered structure, water	[2, 9, 21, 22]
		1640-1611	*β-sheets*	
		1640-1621	parallel β-sheets, intramolecular	[2, 9, 19, 21, 22]
		1620-1611	anti-parallel β-sheets, intermolecular	[2]
		1610-1580	side chains	[19, 21-23]
4: Amide II	1600-1480	1600-1586	anti-parallel β-sheets	[24, 25]

ROI	Deconvoluted range [cm^{-1}]	Spectral range [cm^{-1}]	Assignment	Reference
		1585-1563	β-turns	[24-26]
		1562-1546	α-helix	[2, 24, 26, 27]
		1545-1543	P_{II}-helix	[28]
		1535-1531	parallel β-sheets	[23, 26, 28]
		1530-1528	β-turns	[26, 27]
		1530-1520	random coils	[9, 24, 25]
		1520	*water combination bond*	[3]
		1519-1510	tyrosine	[2, 9, 22, 23]
		1505-1485	parallel β-sheets	[24, 25]

Table S4. Band assignment for deconvoluted regions of interest in Raman spectra (Figure S1).

ROI	Deconvoluted range [cm^{-1}]	Spectral range [cm^{-1}]	Assignment	Reference
1: Amide I	1700-1600	1699-1681	β-turn	[29-32]
		1680-1665	anti-parallel β-sheet OR β-turn	[29, 30, 32-35]
		1665-1660	random coil	[29]
		1660-1640	α-helix	[29-31, 34-36]
		1650-1640	water, disordered structure	[1, 30]
		1639-1620	parallel β-sheet	[29, 30, 34-36]
		1619-1602	amino acid chains	[32]
		1618-1615	*aromatic side chains:* tyrosine, phenylalanine, tryptophan (ν-ring)	[30, 33, 37, 38]
		1606-1602	*aromatic side chains:* tyrosine, phenylalanine, tryptophan (ν-ring)	[30, 33, 34, 37, 39]
2: Amide III	1425-1150	1430-1400	$\nu_s(COO^-)$: aspartic and glutamic acid	[40]
		1398	$\delta(CH_2)$	[38]
		1395-1385	$\delta_s(CH_3)$	[39]
		1370	ν(ring) and ν (CN) in guanine and cytosine	[39]
		1365-1359	tryptophan	[33]
		1341-1339	$\delta(CH_3, CH_2)$: wagging and scissoring in collagen	[41]
		1337	$\delta(CH_3, CH_2)$: twisting mode in collagen	[38]
		1320-1280	amide III: α-helix	[33, 36, 37, 39, 42]
		1275-1242	amide III: disordered structure	[36, 37, 42]
		1241-1220	amide III: β-sheet	[33, 36]
		1210-1208	$\nu(C\text{-}C_6H_5)$ in tyrosine, phenylalanine, tryptophan	[33, 39]
		1177	tryptophan, phenylalanine	[33, 38]
		1174-1169	tyrosine	[38]
		1157	sphingomyelin	[43]
		1155	ν(C-C), δ(C-OH)	[34]
		1128-1124	$\nu(C\text{-}C)_{skeletal}$: acyl backbone in lipid (trans conf.)	[43]
		1130, 1060	$\nu(C\text{-}C)_{skeletal}$	[38]
		1099, 1095	ν(C-N)	[38]

ROI	Deconvoluted range [cm^{-1}]	Spectral range [cm^{-1}]	Assignment	Reference
3: Lower Fingerprint Region	3a: 990-800	983	ν(C-C-N, C-C); proline	[44]
		975	proline/hydroxyproline	[45]
		970	ν(S-S)	[46]
		961	ν(C-C) of residue	[46]
		960	ν(C-C)$_{skeletal}$	[46]
		959-956, 950	Proline	[44]
		940-936	ν(C-C) of protein backbone	[35, 46]
		922-918	ν(C-C) of proline	[35, 46]
		918	ν(C-COO$^-$) of hydroxyproline	[44]
		917	ν(C-COO$^-$) of proline	[44]
		913	hydroxyproline	[47]
		900-890	proline	[44]
		886-884	ν(C-O-C)$_{skeletal}$	[40]
		885-880	ν(ring) of tryptophan	[48]
		879-877	ν(C-C) of hydroxyproline, δ(ring) of tryptophan	[38, 44]
		877-875	ν(C-C)$_{ring}$ of hydroxyproline	[49]
		873	ν(C-C) of hydroxyproline, δ(ring) of tryptophan	[38]
		870-868	proline	[48, 50]
		866	tryptophan	[51]
		862	tyrosine	[52]
		857-854	ν(C-C)$_{ring}$ of proline	[46, 53]
		850	ν(C-C) of hydroxyproline	[44]
		839	proline	[44]
		828-826	out of plane breathing of tyrosine	[41]
		822-821	ν(C-C) of protein backbone	[54]
		817-813	*ν(C-O-C) of crosslinks*	
		817	ν(C-C)$_{skeletal}$	[53]
		815	ν(C-O-C) of glucosyl-galactosyl-hydroxylysine crosslink	[35]
	3b: 800-720	790	δ(COO-) of proline	[44]
		775	ν(C-C)$_{skeletal}$ of proline	[46]
		760-745	ν(C-C) of tryptophan	[33, 39, 55, 56]

ROI	Deconvoluted range [cm^{-1}]	Spectral range [cm^{-1}]	Assignment	Reference
		756	δ(COO-) of hydroxyproline	[44]
		745-700	ν(C-S)$_{trans}$	[37]
		728-727	proline	[48]
		726	ν(C-S) of protein	[48]
	3c: 710-650	703	δ(COO-) of glycine	[46]
		696	hydroxyproline, δ,ω(COO-)	[44]
		689	δ(COO-) of glycine	[46]
		680	proline, ν(C-C)$_{skeletal}$	[46]
		669	ν(C-S) of cytosine	[41]
		662	ν(C-S) of cystine, in COL1	[38]
	3d: 590-470	577	tryptophan	[48]
		562	phenylalanine	[57]
		545-540	tgt(-S-S-), trans-gauche-trans	[58, 59]
		530-523	ggt(-S-S-), gauche-gauche-trans	[58, 59]
		514-487	ggg(-S-S-), gauche-gauche-gauche	[58, 59]
		470	proline	[57]

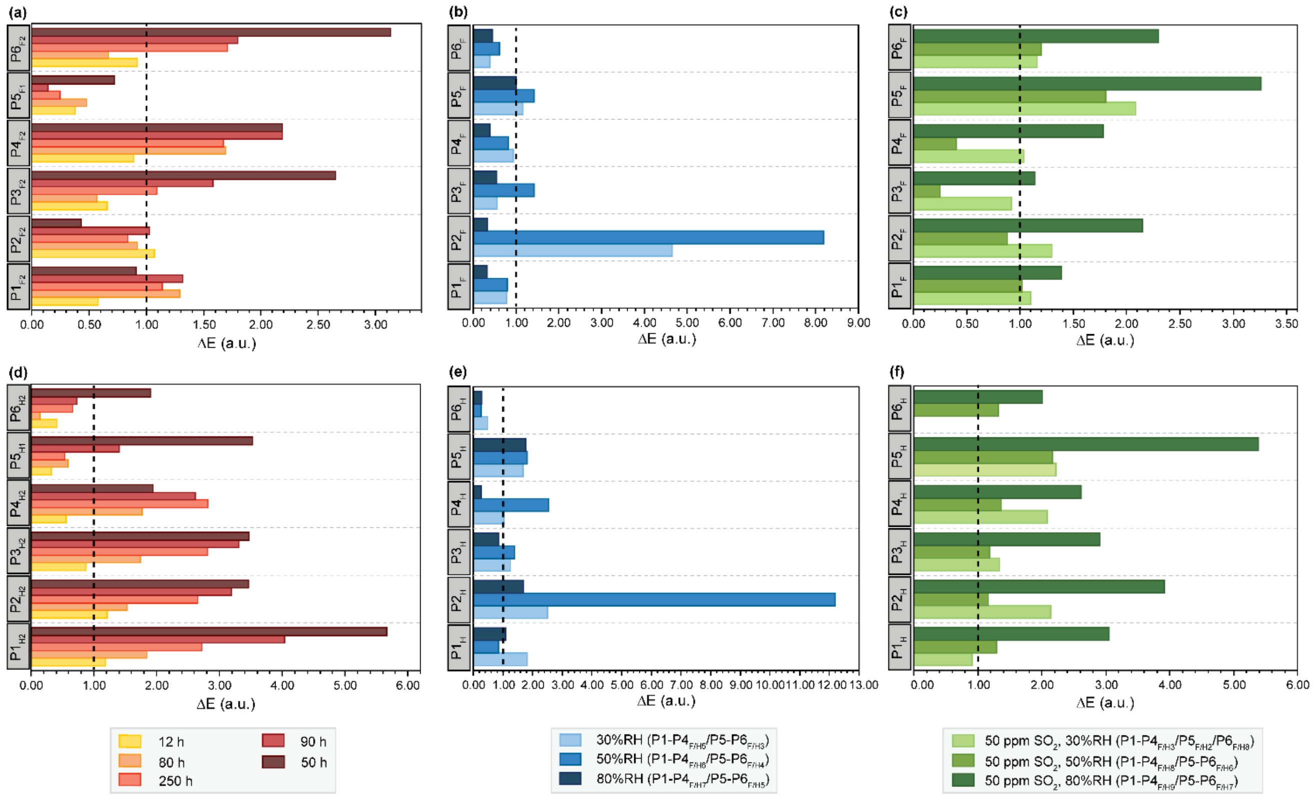

Figure S2. Representation of the color variation ΔE* after exposure of the samples' flesh sides to (a) light, (b) humidity, and (c) SO_2 and the hair sides to (d) light, (e) humidity and (f) SO_2. Vertical dashed line marks a threshold of 1 and the perceptibility by the human eye.

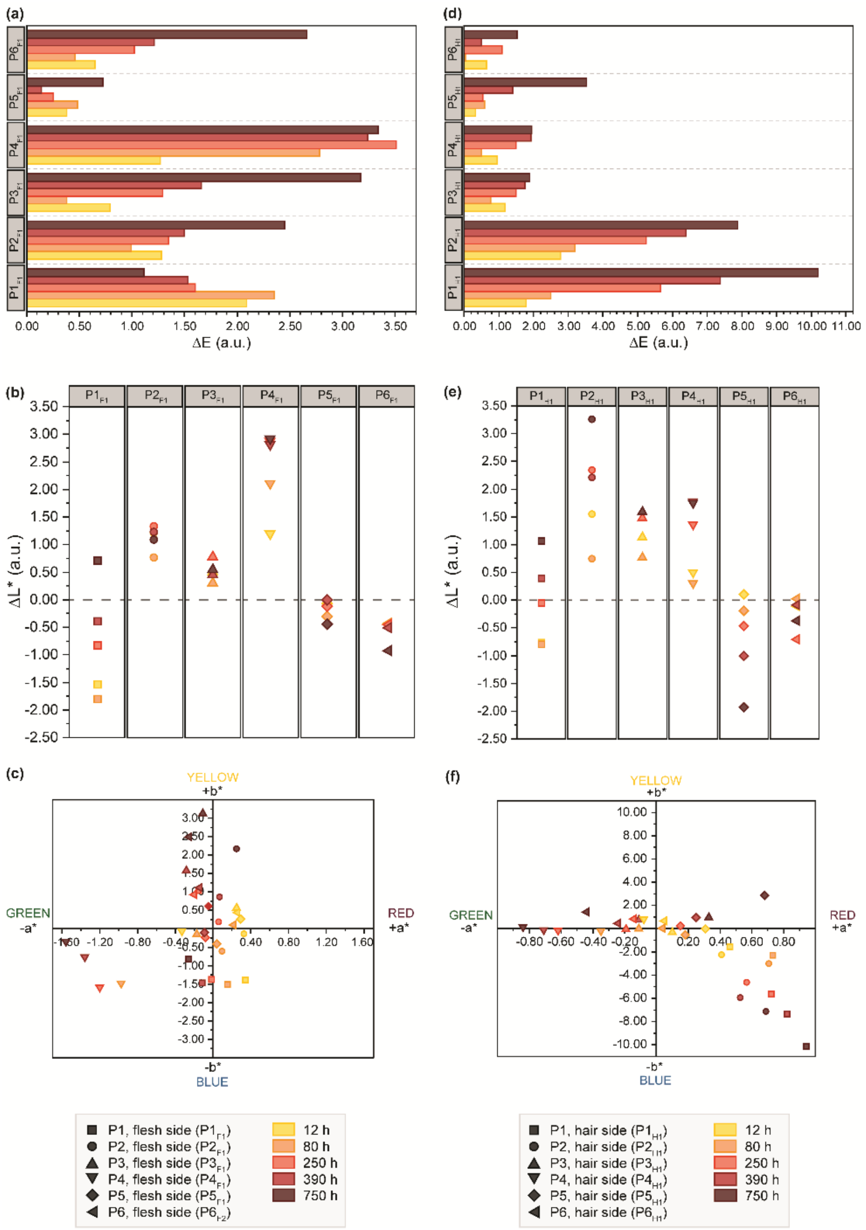

Figure S3. Results of colorimetric measurements after exposure of flesh and hair sides of P1-P6 to light. Changes of (a) and (d) color variation ΔE*, (b) and (e) brightness ΔL* and (c) and (f) single color coordinates for a second batch of light-aged samples.

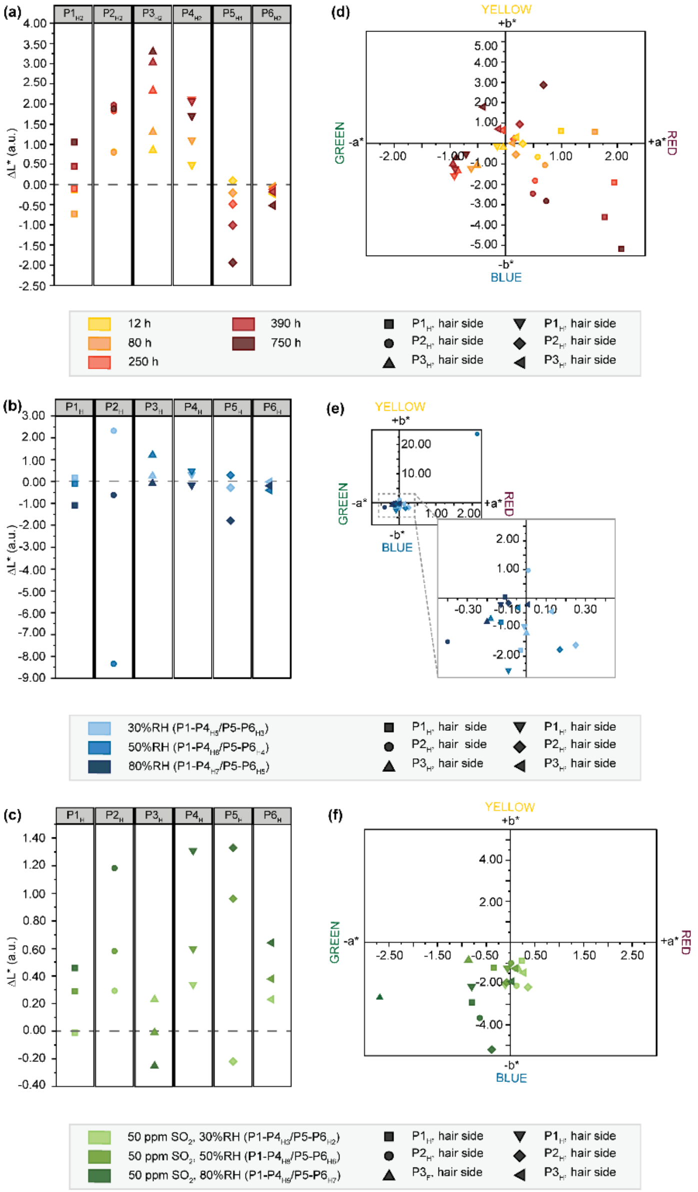

Figure S4. Results of colorimetric measurements after exposure of the hair sides of P1-P6 to light, humidity and SO_2. Changes in brightness ΔL* after exposure to (a) light, (b) humidity and (c) SO_2. Changes of the single color coordinates a* and b* after exposure to (d) light, (e) humidity and (f) SO_2.

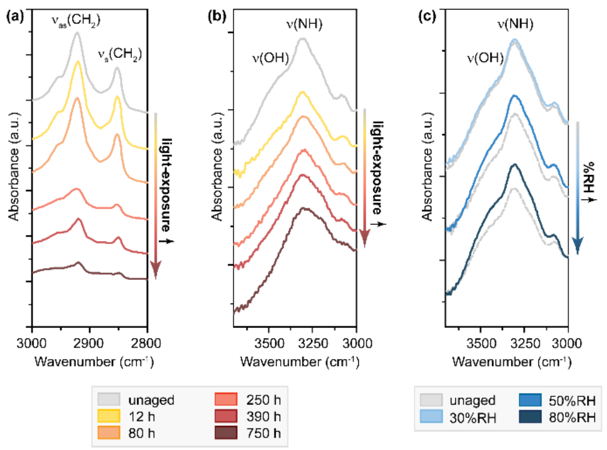

Figure S5. Changes in µ-ATR/FTIR spectra between 3600-2800 cm^{-1} of light- and humidity-exposed parchment. Exposure to light results in a decrease of band intensities of the (a) CH-stretching bands ($v_{as}(CH_2)$ and $v_s(CH_2)$) and (b) the band envelope centered at 3300 cm^{-1}. (c) Increase of band intensities (centered at 3300 cm^{-1}) as a result of increasing humidity levels.

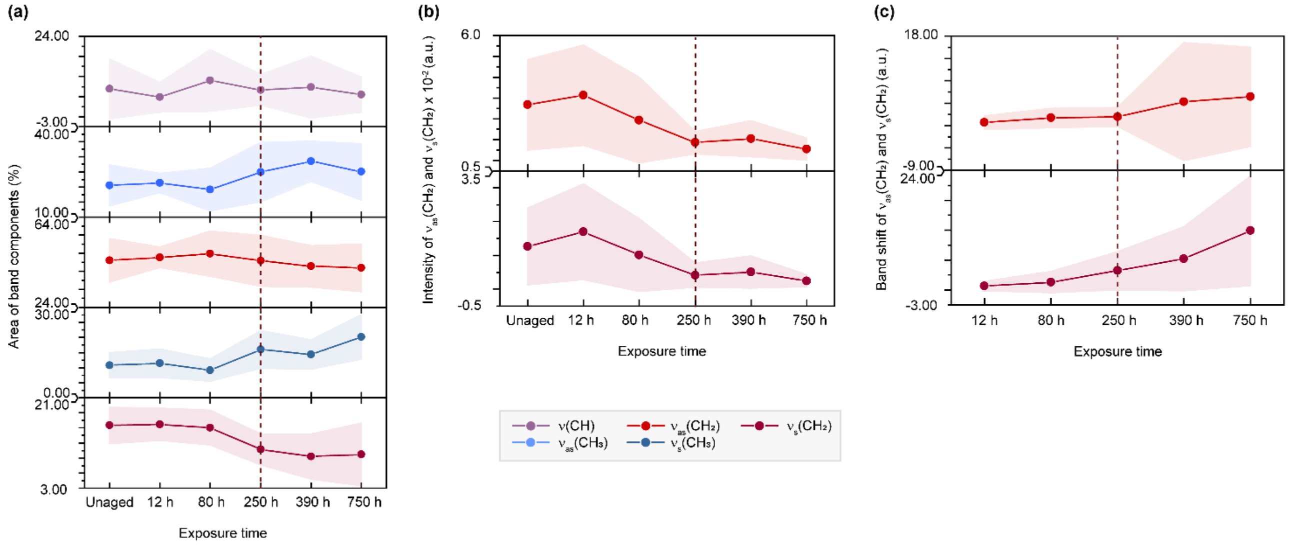

Figure S6. μ-ATR/FTIR spectra between 3000-2800 cm^{-1} of light exposed hair side of parchment. (a) Band areas for ν(CH), $\nu_{as}(CH_2)$ and $\nu_s(CH_2)$, $\nu_{as}(CH_3)$ and $\nu_s(CH_3)$ over 750 h of exposure expressed as percentage from the band envelope area (--- 250 h turning point). (b) S-shaped functions observed for intensities of $\nu_{as}(CH_2)$ at approx. 2920 cm^{-1} and $\nu_s(CH_2)$ at approx. 2850 cm^{-1}. (c) Blue-shift with progressing exposure time of $\nu_{as}(CH_2)$ and $\nu_s(CH_2)$ bands (in the band envelope). (b)-(d) represent averaged values for the hair side of all parchment samples exposed to light.

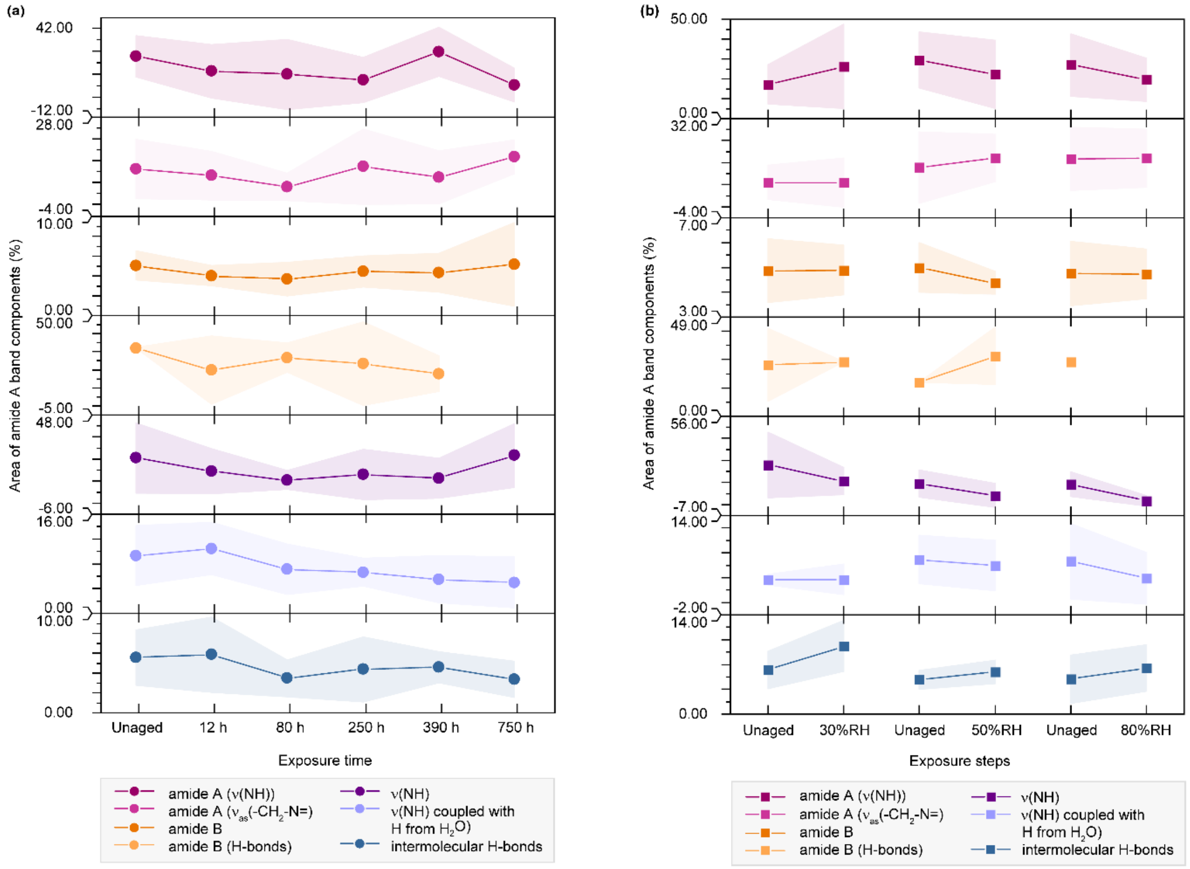

Figure S7. µ-ATR/FTIR spectra between 3600-3000 cm^{-1} of (a) light- and (b) humidity-exposed flesh side of parchment. Band areas of amide A, B and H-bound water-related bands after exposure to (a) light and (b) humidity. Represented averaged values for the flesh side of all parchment samples exposed to (a) light and (b) humidity.

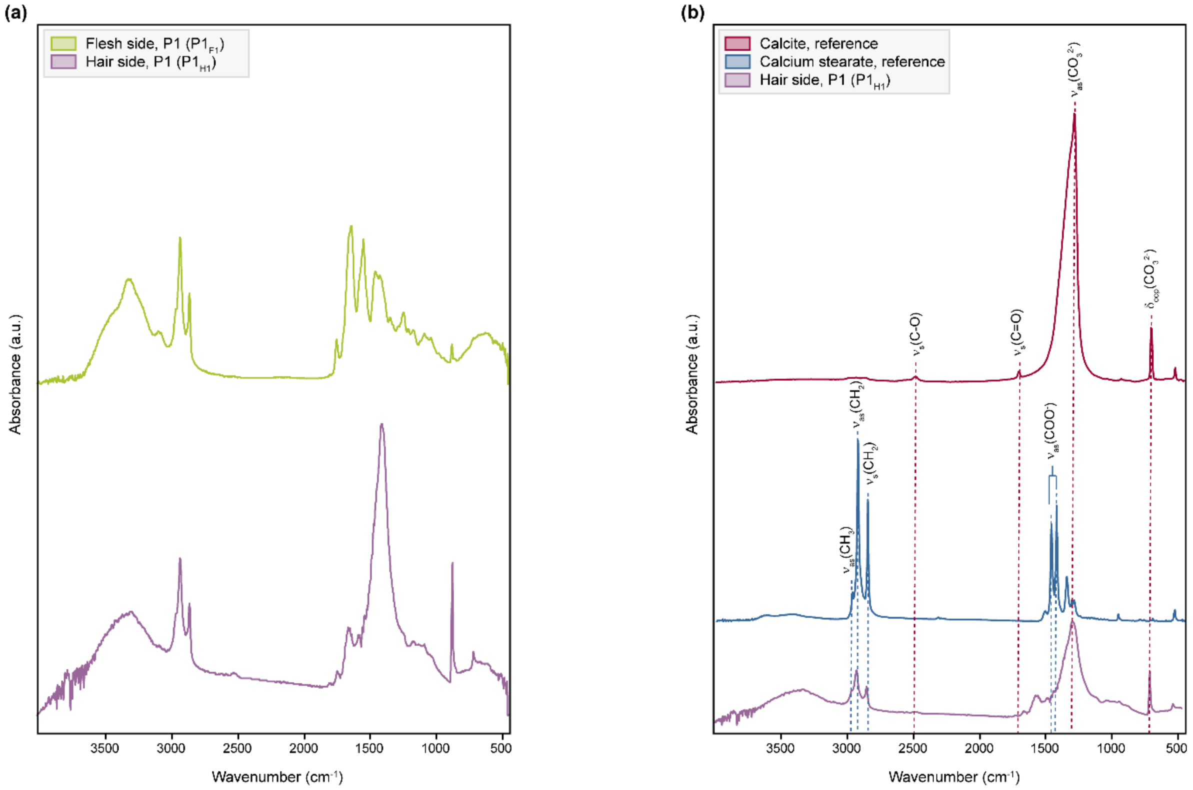

Figure S8. μ-ATR/FTIR spectra of parchment P1. (a) Comparison between hair and flesh side. (b) Comparison of hair side with calcite and calcium stearate reference spectra.

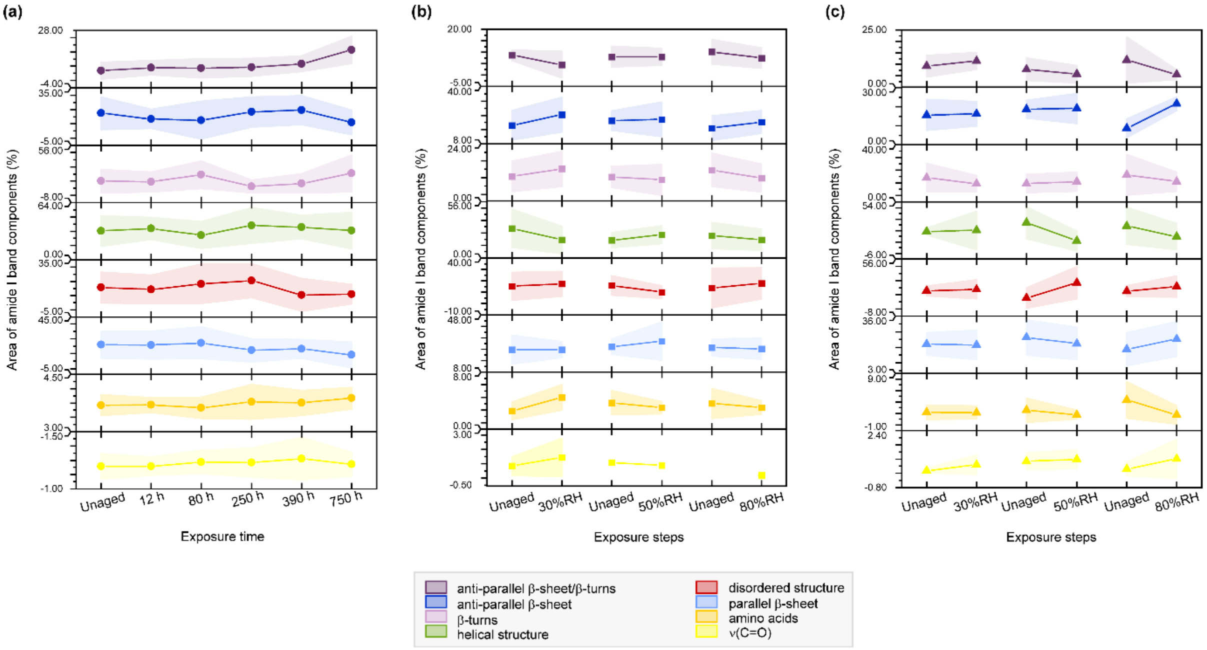

Figure S9. μ-ATR/FTIR spectra between 1720-1580 cm^{-1} of light-, humidity- and SO_2- exposed hair side of parchment. Averaged band areas for the amide I band components after (a) light, (b) humidity and (c) SO_2-expsure as percentage from the band envelope.

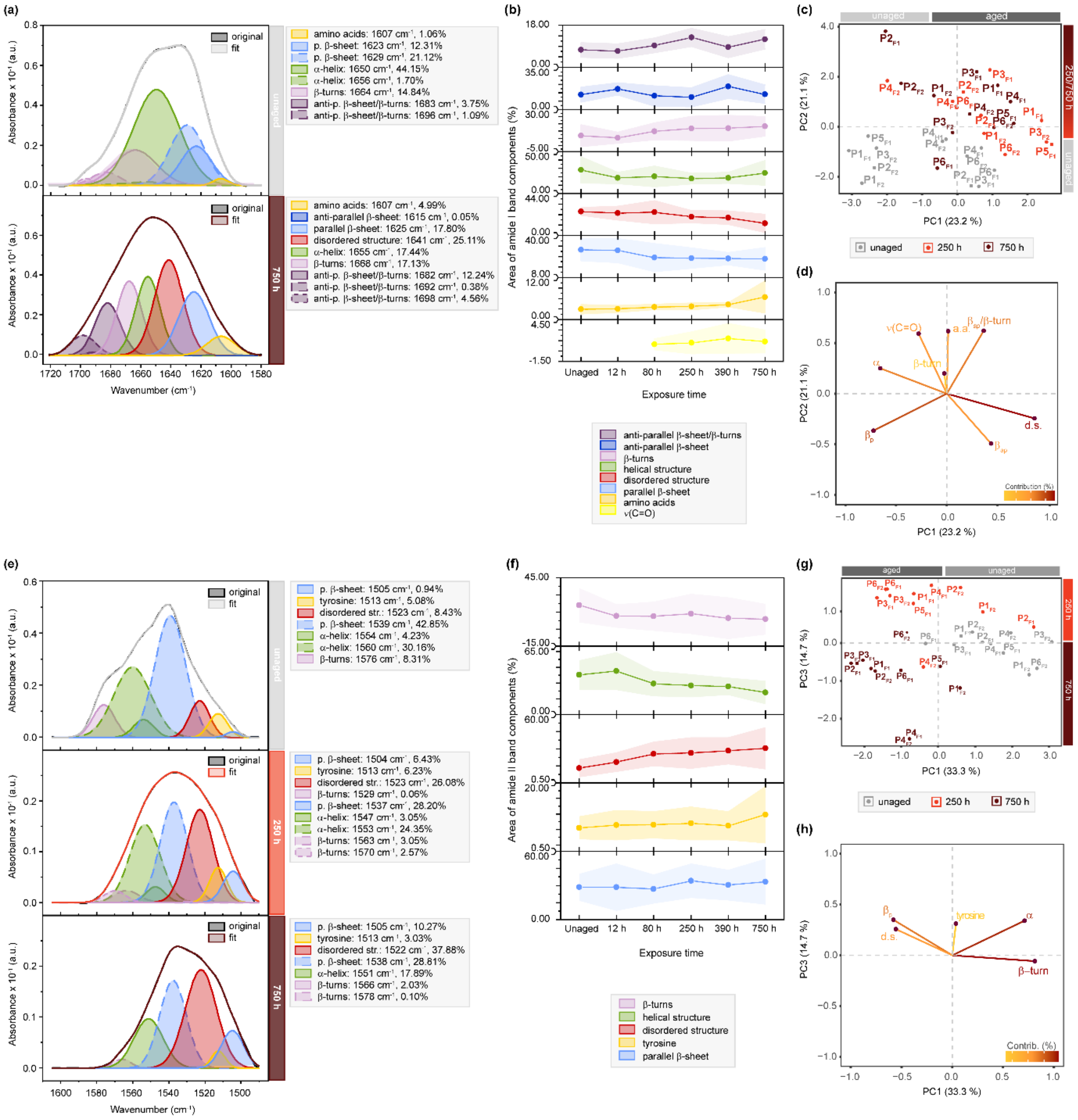

Figure S10. μ-ATR/FTIR spectra between 1720-1580 cm^{-1} (amide I) and 1590-1480 cm^{-1} (amide II) of light-exposed flesh side of parchment. (a) Exemplary band deconvolution of the amide I band envelope of unaged and 750 h-aged sample $P1_{F1}$. (b) Band areas for amide I band components as percentage of the band envelope. Separation of unaged and aged samples observed in (c) score plots and contributing variables in (d) loading plots. (e) Exemplary band deconvolution of the amide II band envelope of unaged and 750 h-aged sample $P1_{F1}$. (f) Band areas for amide II band components as percentage of the band envelope. Separation of unaged and aged samples observed in (g) score plots and contributing variables in (h) loading plots.

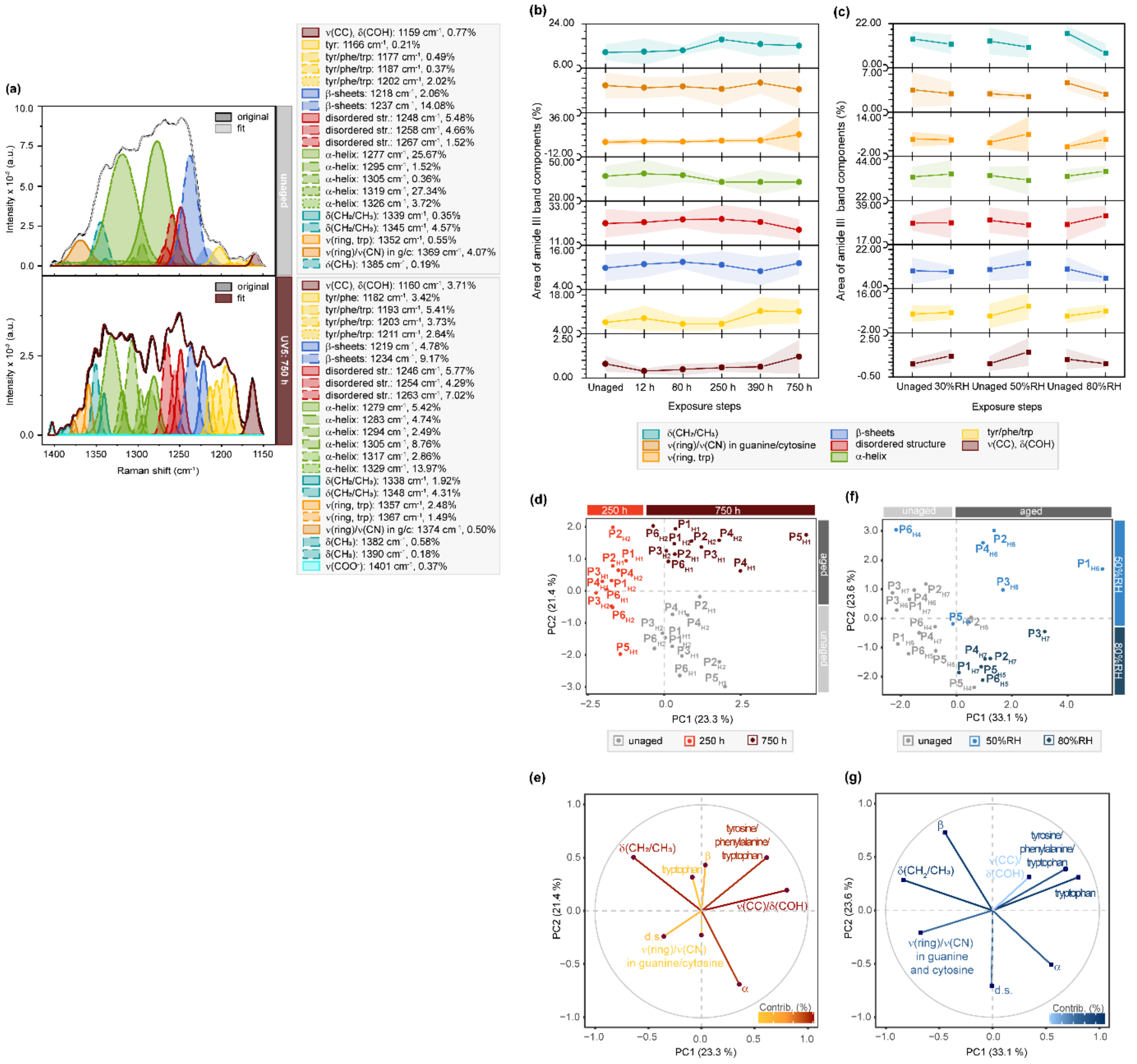

Figure S11. μ-Raman spectra between 1425-1150 cm^{-1} of light- and humidity-exposed hair side of parchment. (a) Exemplary band deconvolution of amide III band envelope of unaged and 750 h-aged sample P5$_{H1}$. Band areas for amide III band components after exposure to (b) light and (c) 30%RH/50%RH/80%RH as percentage of the band envelope. Separation of unaged and aged samples observed in score plots and contributing variables in the loading plots (d-e) after light- and (f-g) humidity-exposure.

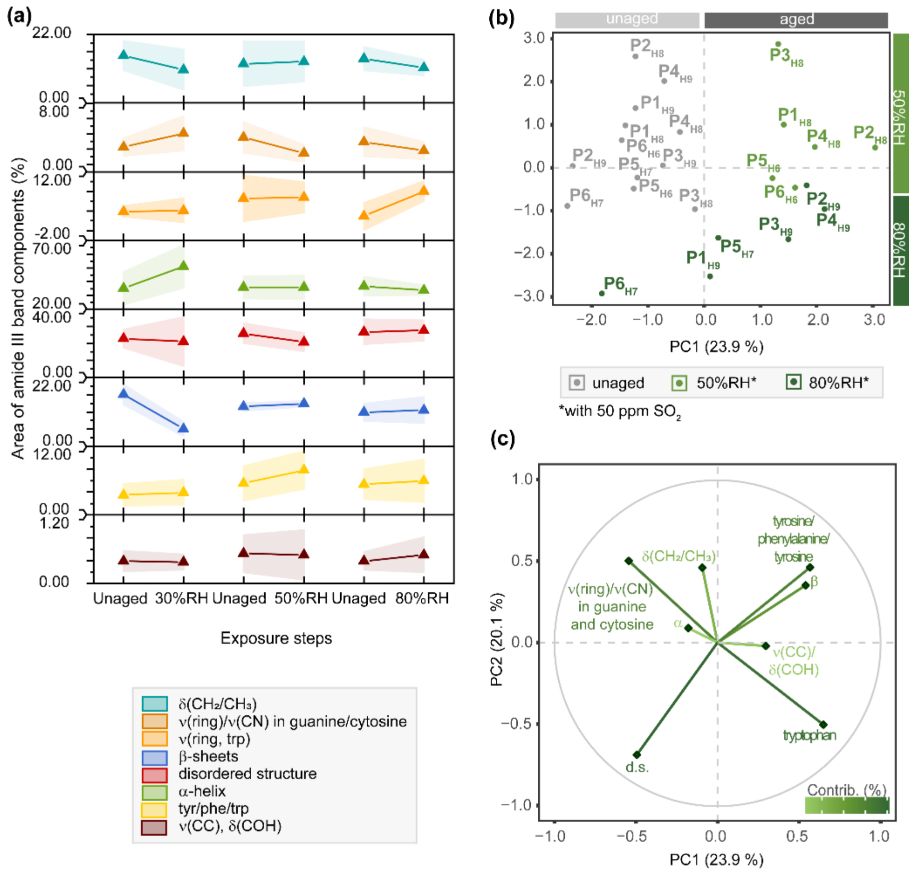

Figure. S12. µ-Raman spectra between 1425-1150 cm^{-1} of SO_2-exposed hair side of parchment. (a) Band areas for amide III band components after exposure to 50 ppm SO_2 and 30%RH/50%RH/80%RH as percentage of the band envelope. Separation of unaged and aged samples observed in (b) the score plot and contributing variables (c) in the loading plots.

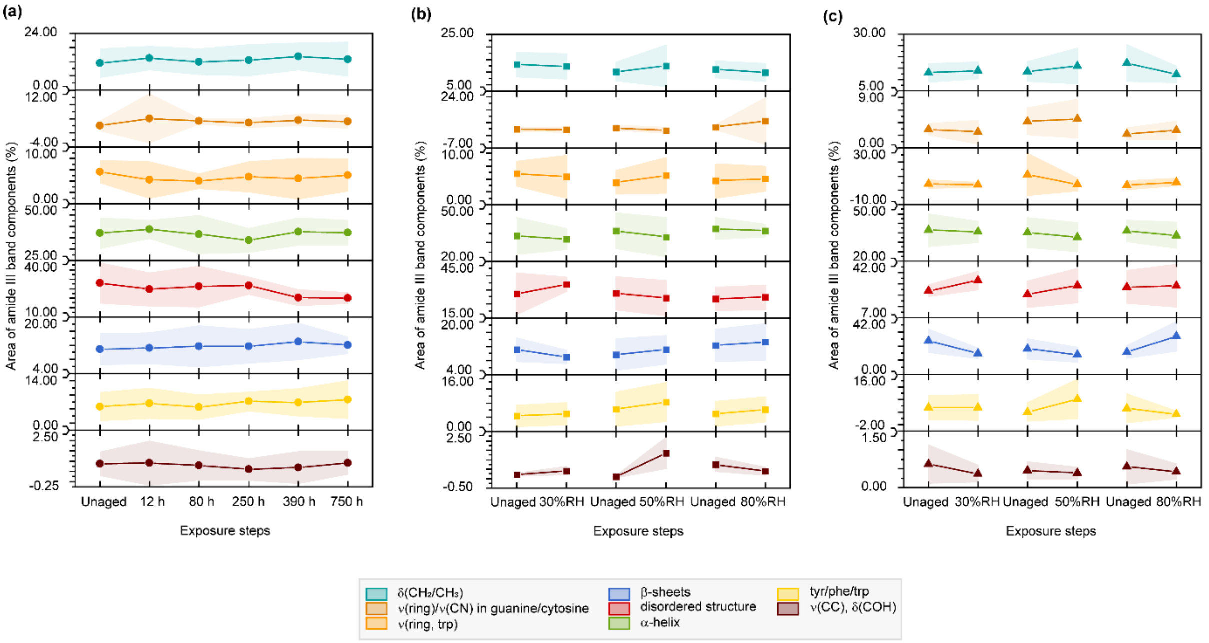

Figure S13. μ-Raman spectra between 1425-1150 cm^{-1} after exposure to light, humidity and SO2 of flesh side of parchment. Band areas for amide III band components after exposure to (a) light, 30%RH/50%RH/80%RH (b) without and (c) with 50 ppm SO_2 as percentage of the band envelope.

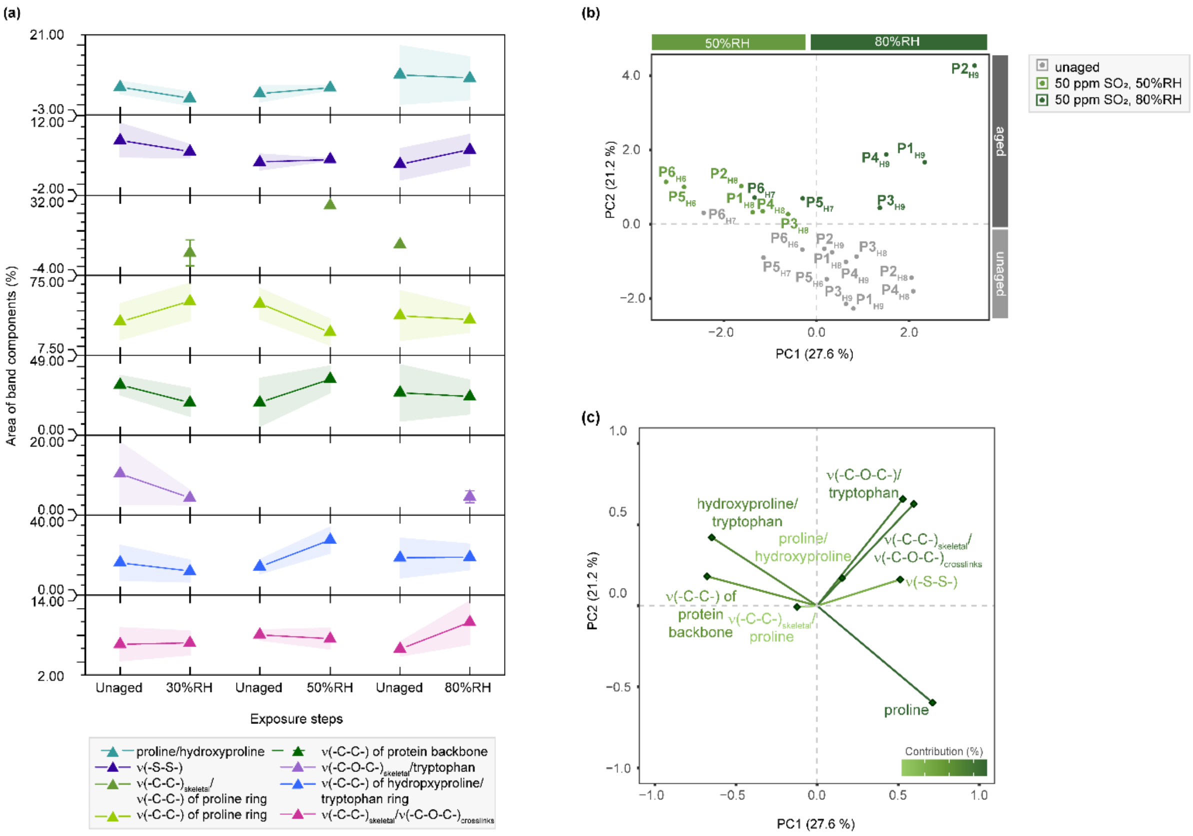

Figure S14. μ-Raman spectra between 1000-800 cm^{-1} of SO_2-exposed hair side of parchment. (a) Band areas of the respective band components after exposure to 30%RH/50%RH/80%RH and 50 ppm SO_2 as percentage from the band envelope area. Separation of unaged and aged samples observed in (b) score plots and contributing variables in (c) the loading plots.

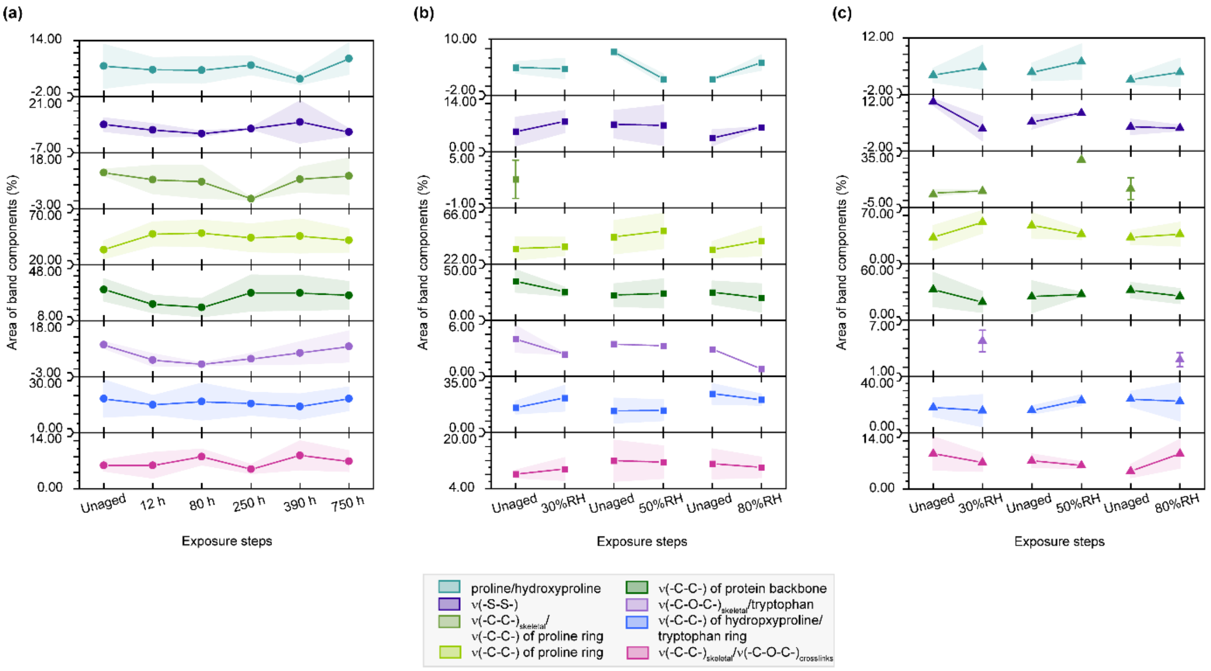

Figure S15. μ-Raman spectra between 1000-800 cm^{-1} of light-, humidity- and SO_2-exposed flesh side of parchment. Band areas of the respective band components after exposure to (a) light, 30%RH/50%RH//80%RH (b) without and (c) with 50 ppm SO_2 as percentage from the band envelope area.

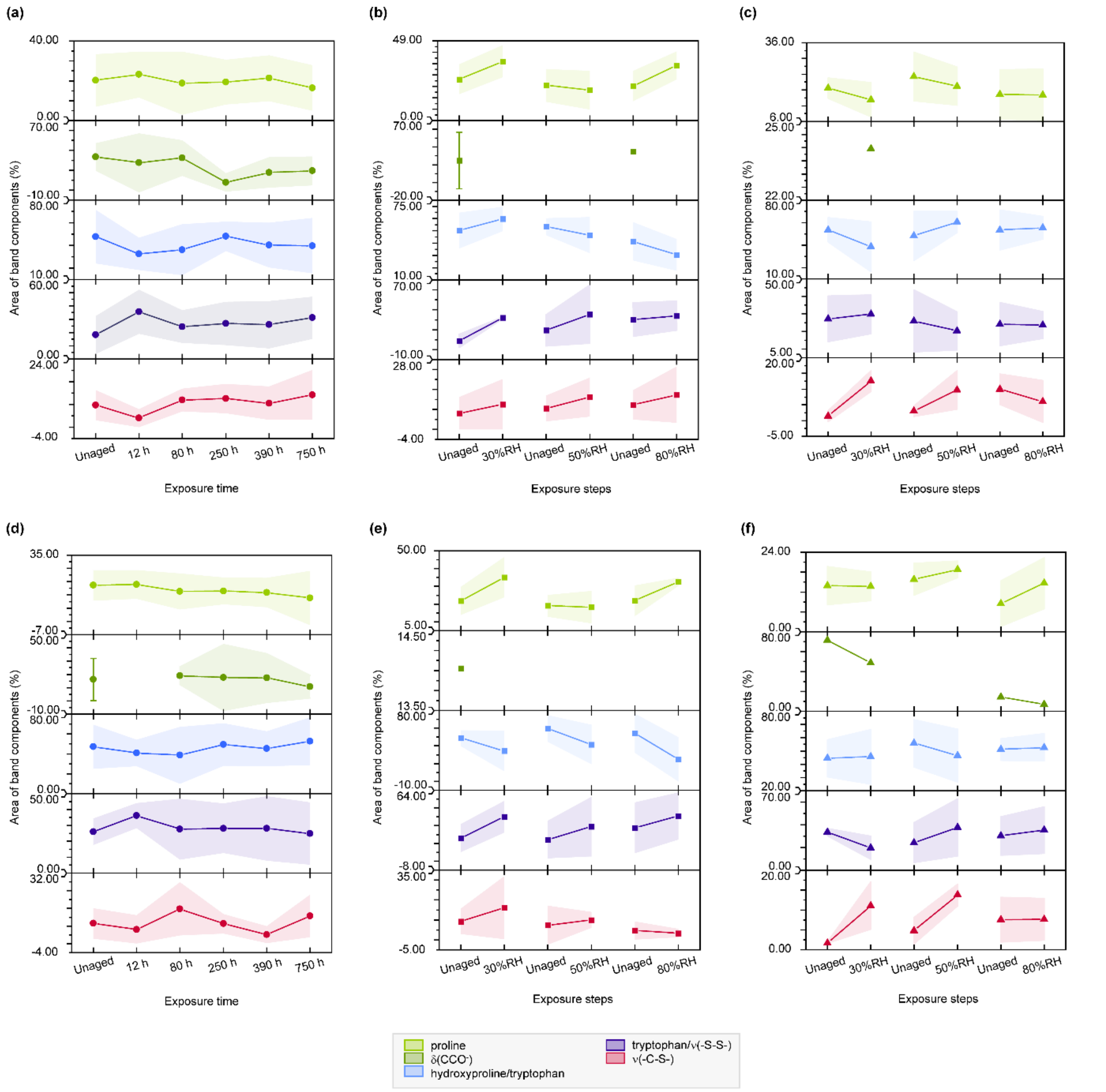

Figure S16. μ-Raman spectra between 800-720 cm^{-1} of light, humidity and SO_2-exposed of parchment. Band areas of the respective band components after exposure to (a) light, 30%RH/50%RH//80%RH (b) without and (c) with 50 ppm SO_2 as percentage from the band envelope area of flesh sides. (d)-(f) of exposed hair sides.

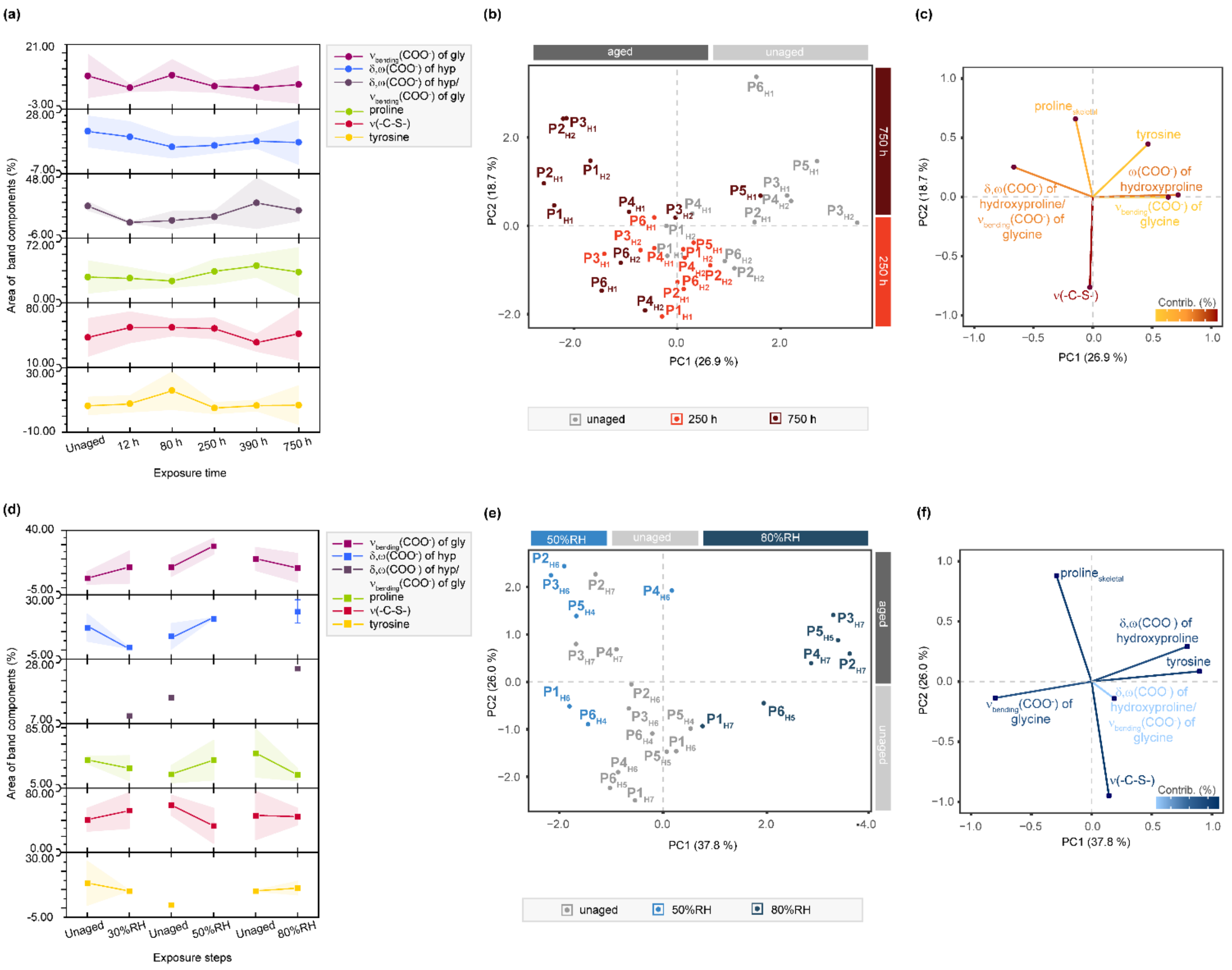

Figure S17. μ-Raman spectra between 710-650 cm^{-1} of light- and humidity-exposed hair side of parchment. (a) Band areas of the respective band components after light-exposure over 750 h and (d) RH-exposure to 30%RH/50%RH/80%RH as percentage from the band envelope area. Separation of unaged and aged samples observed in score plots and contributing variables in the loading plots (b)-(c) after light-exposure and (e)-(f) after RH-aging.

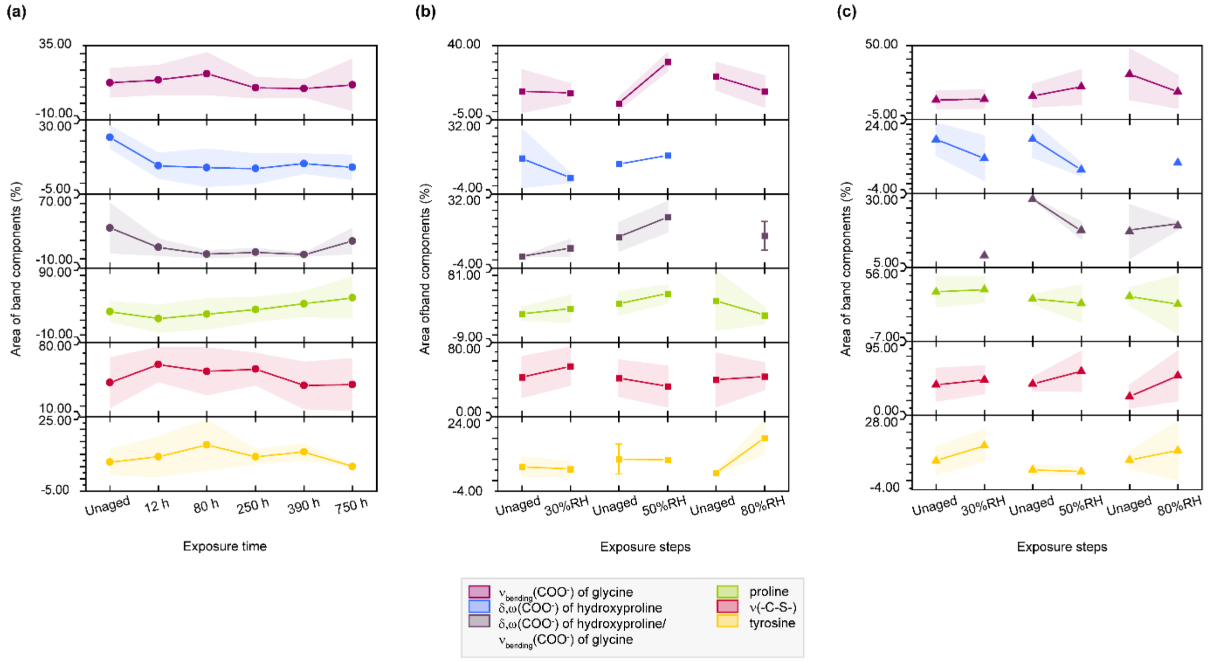

Figure S18. μ-Raman spectra between 710-650 cm^{-1} of light, humidity and SO_2-exposed flesh side of parchment. Band areas of the respective band components after exposure to (a) light, 30%RH/50%RH//80%RH (b) without and (c) with 50 ppm SO_2 as percentage from the band envelope area.

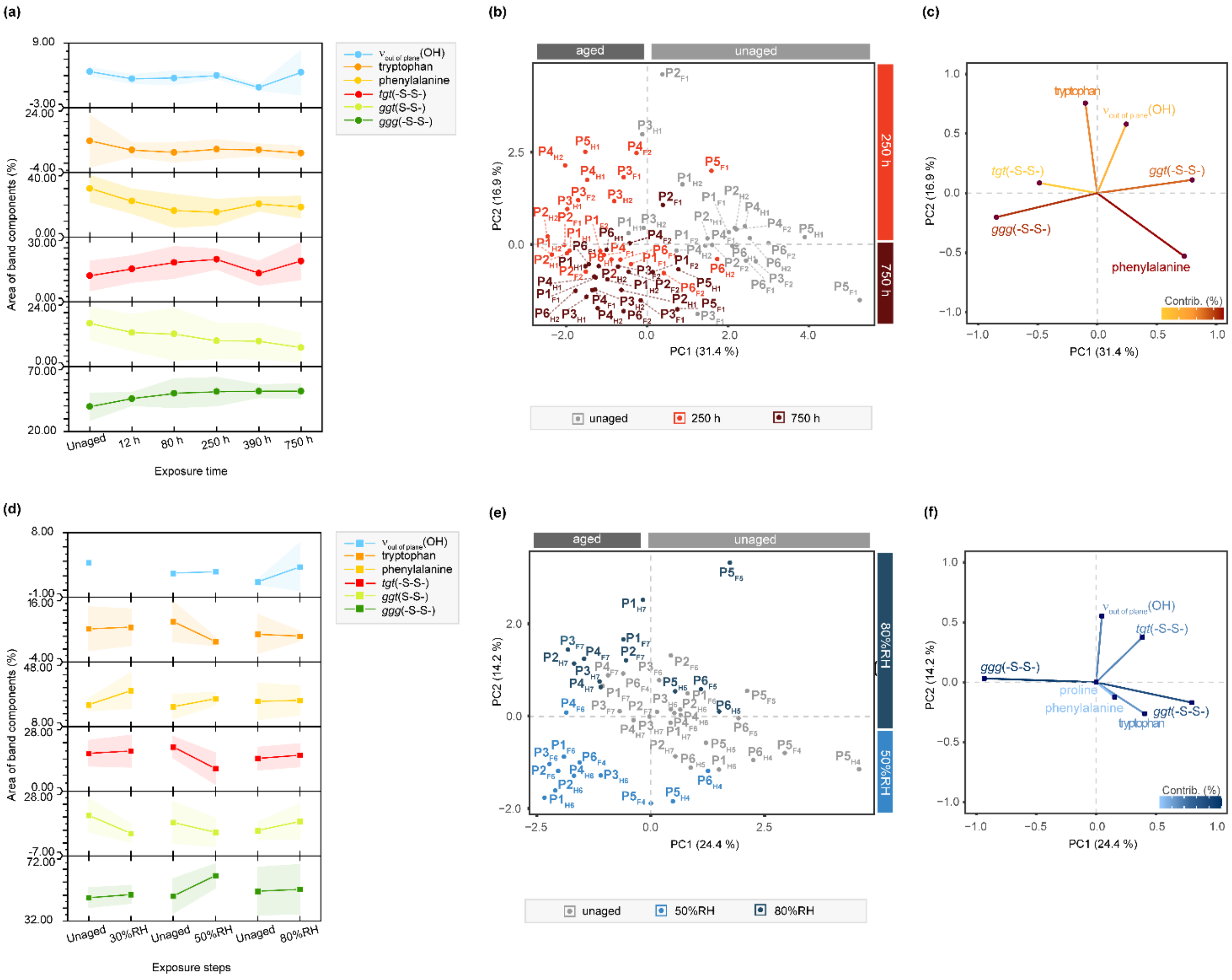

Figure S19. μ-Raman spectra between 590-470 cm^{-1} of light- and humidity-exposed hair side of parchment. (a) Band areas of the respective band components after light-exposure over 750 h and (d) RH-exposure to 30%RH/50%RH/80%RH as percentage from the band envelope area. Separation of unaged and aged samples observed in score plots and contributing variables in the loading plots (b)-(c) after light-exposure and (e)-(f) after RH-aging.

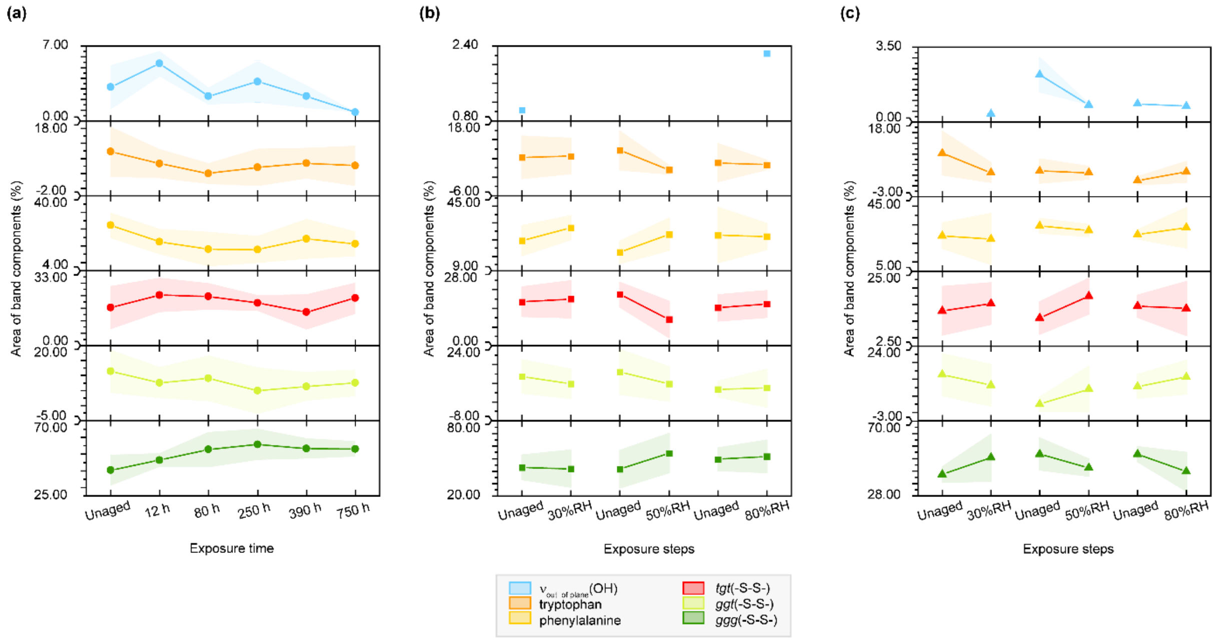

Figure S20. μ-Raman spectra between 590-470 cm^{-1} of light, humidity and SO_2-exposed parchment. Band areas of the respective band components after exposure to (a) light, 30%RH/50%RH//80%RH (b) without and (c) with 50 ppm SO_2 as percentage from the band envelope area of flesh sides. (d)-(f) of exposed hair sides.

References

[1] Barique, M. A.; Tsuchida, E.; Ohira, A.; Tashiro, K., Effect of elevated temperatures on the states of water and their correlation with the proton conductivity of Nafion. ACS omega **2018,** 3 (1), 349-360.

[2] Olsztyńska-Janus, S.; Pietruszka, A.; Kiełbowicz, Z.; Czarnecki, M. A., ATR-IR study of skin components: Lipids, proteins and water. Part I: Temperature effect. Spectrochimica Acta Part A: Molecular and Biomolecular Spectroscopy **2018,** 188, 37-49.

[3] Lucassen, G. W.; Van Veen, G. N.; Jansen, J. A., Band analysis of hydrated human skin stratum corneum attenuated total reflectance Fourier transform infrared spectra in vivo. Journal of biomedical optics **1998,** 3 (3), 267-280.

[4] Wu, J. G.; Xu, Y. Z.; Sun, C. W.; Soloway, R. D.; Xu, D. F.; Wu, Q. G.; Sun, K. H.; Weng, S. F.; Xu, G. X., Distinguishing malignant from normal oral tissues using FTIR fiber-optic techniques. Biopolymers: Original Research on Biomolecules **2001,** 62 (4), 185-192.

[5] Dovbeshko, G. I.; Gridina, N. Y.; Kruglova, E. B.; Pashchuk, O. P., FTIR spectroscopy studies of nucleic acid damage. Talanta **2000,** 53 (1), 233-246.

[6] Schulz, H.; Baranska, M., Identification and quantification of valuable plant substances by IR and Raman spectroscopy. Vibrational Spectroscopy **2007,** 43 (1), 13-25.

[7] Smith, R.; Rehman, I., Fourier transform Raman spectroscopic studies of human bone. Journal of Materials Science: Materials in Medicine **1994,** 5, 775-778.

[8] Movasaghi, Z.; Rehman, S.; ur Rehman, D. I., Fourier transform infrared (FTIR) spectroscopy of biological tissues. Applied Spectroscopy Reviews **2008,** 43 (2), 134-179.

[9] Rabotyagova, O. S.; Cebe, P.; Kaplan, D. L., Collagen Structural Hierarchy and Susceptibility to Degradation by Ultraviolet Radiation. Mater Sci Eng C Mater Biol Appl **2008,** 28 (8), 1420-1429.

[10] Eckel, R.; Huo, H.; Guan, H.-W.; Hu, X.; Che, X.; Huang, W.-D., Characteristic infrared spectroscopic patterns in the protein bands of human breast cancer tissue. Vibrational Spectroscopy **2001,** 27 (2), 165-173.

[11] Dovbeshko, G.; Chegel, V.; Gridina, N. Y.; Repnytska, O.; Shirshov, Y.; Tryndiak, V.; Todor, I.; Solyanik, G., Surface enhanced IR absorption of nucleic acids from tumor cells: FTIR reflectance study. Biopolymers: Original Research on Biomolecules **2002,** 67 (6), 470-486.

[12] Yang, Y.; Sulé-Suso, J.; Sockalingum, G. D.; Kegelaer, G.; Manfait, M.; El Haj, A. J., Study of tumor cell invasion by Fourier transform infrared microspectroscopy. Biopolymers: Original Research on Biomolecules **2005,** 78 (6), 311-317.

[13] Paluszkiewicz, C.; Kwiatek, W. M., Analysis of human cancer prostate tissues using FTIR microspectroscopy and SRIXE techniques. Journal of Molecular Structure **2001,** 565, 329-334.

[14] Flach, C. R.; Moore, D. J., Infrared and Raman imaging spectroscopy of ex vivo skin. International Journal of Cosmetic Science **2013,** 35 (2), 125-135.

[15] Saeed, A.; Raouf, G. A.; Nafee, S. S.; Shaheen, S. A.; Al-Hadeethi, Y., Effects of very low dose fast neutrons on cell membrane and secondary protein structure in rat erythrocytes. PLoS one **2015,** 10 (10), e0139854.

[16] Arrondo, J. L. R.; Goni, F. M., Infrared studies of protein-induced perturbation of lipids in lipoproteins and membranes. Chemistry and physics of lipids **1998,** 96 (1-2), 53-68.

[17] Mendelsohn, R.; Flach, C. R.; Moore, D. J., Determination of molecular conformation and permeation in skin via IR spectroscopy, microscopy, and imaging. Biochimica et Biophysica Acta (BBA)-Biomembranes **2006,** 1758 (7), 923-933.

[18] Coates, J., Interpretation of infrared spectra, a practical approach. 2000.

[19] Barth, A., Infrared spectroscopy of proteins. Biochimica et Biophysica Acta (BBA) - Bioenergetics **2007,** 1767 (9), 1073-1101.

[20] Kavanagh, G. M.; Clark, A. H.; Ross-Murphy, S. B., Heat-induced gelation of globular proteins: part 3. Molecular studies on low pH β-lactoglobulin gels. International Journal of Biological Macromolecules **2000,** 28 (1), 41-50.

[21] Chadefaux, C.; Le Hô, A.-S.; Bellot-Gurlet, L.; Reiche, I., Curve-fitting Micro-ATR-FTIR studies of the amide I and II bands of type I collagen in archaeological bone materials. e-PRESERVATION Science **2009,** 6, 129-137.

[22] Kreuzer, M.; Dučić, T.; Hawlina, M.; Andjelic, S., Synchrotron-based FTIR microspectroscopy of protein aggregation and lipids peroxidation changes in human cataractous lens epithelial cells. Scientific Reports **2020,** 10 (1), 15489.

[23] Goormaghtigh, E.; Ruysschaert, J.-M.; Raussens, V., Evaluation of the Information Content in Infrared Spectra for Protein Secondary Structure Determination. Biophysical Journal **2006,** 90 (8), 2946-2957.

[24] Adochitei, A.; Drochioiu, G., Rapid Characterization of peptide secondary structure by FT-IR spectroscopy. Revue Roumaine de Chimie **2011,** 56, 783-791.

[25] Abu Teir, M.; Ghithan, J.; Darwish, S.; Abu-hadid, M., Multi-spectroscopic investigation of the interactions between cholesterol and human serum albumin. Journal of Applied Biological Sciences **2012,** 6, 45-55.

[26] Téllez Soto, C. A.; Medeiros-Neto, L. P.; dos Santos, L.; Santos, A. B.; Ferreira, I.; Singh, P.; Canevari, R. A.; Martin, A. A., Infrared and confocal Raman spectroscopy to differentiate changes in the protein secondary structure in normal and abnormal thyroid tissues. Journal of Raman Spectroscopy **2018,** 49 (7), 1165-1173.

[27] Azizova, L. R.; Kulik, T. V.; Palianytsia, B. B.; Zemlyakov, A. E.; Tsikalova, V. N.; Chirva, V. Y., Investigation of chemical transformations of thiophenylglycoside of muramyl dipeptide on the fumed silica surface using TPD-MS, FTIR spectroscopy and ES IT MS. Nanoscale Research Letters **2014,** 9, 1-9.

[28] Mirtič, A.; Grdadolnik, J., The structure of poly-L-lysine in different solvents. Biophysical chemistry **2013,** 175, 47-53.

[29] Sadat, A.; Joye, I. J., Peak fitting applied to fourier transform infrared and raman spectroscopic analysis of proteins. Applied Sciences **2020,** 10 (17), 5918.

[30] Byler, D. M.; Farrell Jr, H. M.; Susi, H., Raman spectroscopic study of casein structure. Journal of Dairy Science **1988,** 71 (10), 2622-2629.

[31] Peters, J.; Park, E.; Kalyanaraman, R.; Luczak, A.; Ganesh, V., Protein secondary structure determination using drop coat deposition confocal raman spectroscopy. Spectroscopy **2016,** 31 (10), 31–39-31–39.

[32] Rivas-Arancibia, S.; Rodríguez-Martínez, E.; Badillo-Ramírez, I.; López-González, U.; Saniger, J. M., Structural changes of amyloid beta in hippocampus of rats exposed to ozone: A Raman spectroscopy study. Frontiers in Molecular Neuroscience **2017,** 10, 137.

[33] Devitt, G.; Rice, W.; Crisford, A.; Nandhakumar, I.; Mudher, A.; Mahajan, S., Conformational evolution of molecular signatures during amyloidogenic protein aggregation. ACS chemical neuroscience **2019,** 10 (11), 4593-4611.

[34] Martinez, M. G.; Bullock, A. J.; MacNeil, S.; Rehman, I. U., Characterisation of structural changes in collagen with Raman spectroscopy. Applied Spectroscopy Reviews **2019,** 54 (6), 509-542.

[35] Ye, H.; Rahul; Kruger, U.; Wang, T.; Shi, S.; Norfleet, J.; De, S., Burn-related Collagen Conformational Changes in ex vivo Porcine Skin using Raman Spectroscopy. Scientific Reports **2019,** 9 (1), 19138.

[36] Voicescu, M.; Ionescu, S.; Nistor, C. L., Spectroscopic study of 3-Hydroxyflavone-protein interaction in lipidic bi-layers immobilized on silver nanoparticles. Spectrochimica Acta Part A: Molecular and Biomolecular Spectroscopy **2017,** 170, 1-8.

[37] Herrero, A. M., Raman spectroscopy for monitoring protein structure in muscle food systems. Critical reviews in food science and nutrition **2008,** 48 (6), 512-523.

[38] Cheng, W. T.; Liu, M. T.; Liu, H. N.; Lin, S. Y., Micro-Raman spectroscopy used to identify and grade human skin pilomatrixoma. Microscopy research and technique **2005,** 68 (2), 75-79.

[39] Pezzotti, G.; Boffelli, M.; Miyamori, D.; Uemura, T.; Marunaka, Y.; Zhu, W.; Ikegaya, H., Raman spectroscopy of human skin: looking for a quantitative algorithm to reliably estimate human age. Journal of Biomedical Optics **2015,** 20 (6), 065008-065008.

[40] Shetty, G.; Kendall, C.; Shepherd, N.; Stone, N.; Barr, H., Raman spectroscopy: elucidation of biochemical changes in carcinogenesis of oesophagus. British journal of cancer **2006,** 94 (10), 1460-1464.

[41] Stone, N.; Kendall, C.; Smith, J.; Crow, P.; Barr, H., Raman spectroscopy for identification of epithelial cancers. Faraday discussions **2004,** 126, 141-157.

[42] Unal, M.; Jung, H.; Akkus, O., Novel Raman spectroscopic biomarkers indicate that postyield damage denatures bone's collagen. Journal of bone and mineral research **2016,** 31 (5), 1015-1025.

[43] Franzen, L.; Windbergs, M., Applications of Raman spectroscopy in skin research—from skin physiology and diagnosis up to risk assessment and dermal drug delivery. Advanced drug delivery reviews **2015,** 89, 91-104.

[44] Cárcamo, J. J.; Aliaga, A. E.; Clavijo, E.; Garrido, C.; Gómez-Jeria, J. S.; Campos-Vallette, M. M., Proline and hydroxyproline deposited on silver nanoparticles. A Raman, SERS and theoretical study. Journal of Raman Spectroscopy **2012,** 43 (6), 750-755.

[45] Wang, S.-s.; Ye, D.-x.; Wang, B.; Xie, C., The expressions of keratins and P63 in primary squamous cell carcinoma of the thyroid gland: an application of raman spectroscopy. OncoTargets and therapy **2020,** 13, 585.

[46] Penteado, S. C. G.; Fogazza, B. P.; Carvalho, C. d. S.; Arisawa, E. A. L.; Martins, M. A.; Martin, A. A.; Martinho, H. d. S., Diagnosis of degenerative lesions of supraspinatus rotator cuff tendons by Fourier transform-Raman spectroscopy. Journal of Biomedical Optics **2008,** 13 (1), 014018-014018-10.

[47] Tellez Soto, C. A.; Pereira, L.; Dos Santos, L.; Rajasekaran, R.; Fávero, P.; Martin, A. A., DFT: B3LYP/3-21G theoretical insights on the confocal Raman experimental observations in skin dermis of healthy young, healthy elderly, and diabetic elderly women. Journal of biomedical optics **2016,** 21 (12), 125002-125002.

[48] Movasaghi, Z.; Rehman, S.; Rehman, I. U., Raman spectroscopy of biological tissues. Applied Spectroscopy Reviews **2007,** 42 (5), 493-541.

[49] Huang, Z.; McWilliams, A.; Lui, H.; McLean, D. I.; Lam, S.; Zeng, H., Near-infrared Raman spectroscopy for optical diagnosis of lung cancer. International journal of cancer **2003,** 107 (6), 1047-1052.

[50] Gniadecka, M.; Wulf, H.; Nymark Mortensen, N.; Faurskov Nielsen, O.; Christensen, D. H., Diagnosis of basal cell carcinoma by Raman spectroscopy. Journal of Raman spectroscopy **1997,** 28 (2-3), 125-129.

[51] Aliaga, A.; Osorio-Román, I.; Leyton, P.; Garrido, C.; Carcamo, J.; Caniulef, C.; Celis, F.; Díaz F, G.; Clavijo, E.; Gómez-Jeria, J., Surface-enhanced Raman scattering study of L-tryptophan. Journal of Raman Spectroscopy: An International Journal for Original Work in all Aspects of Raman Spectroscopy, Including Higher Order Processes, and also Brillouin and Rayleigh Scattering **2009,** 40 (2), 164-169.

[52] Pinheiro, A. L. B.; Santos, N. R. S.; Oliveira, P. C.; Aciole, G. T. S.; Ramos, T. A.; Gonzalez, T. A.; da Silva, L. N.; Barbosa, A. F. S.; Silveira, L., The efficacy of the use of IR laser phototherapy associated to biphasic ceramic graft and guided bone regeneration on surgical fractures treated with wire osteosynthesis: a comparative laser fluorescence and Raman spectral study on rabbits. Lasers in medical science **2013,** 28, 815-822.

[53] Frank, C. J.; McCreery, R. L.; Redd, D. C., Raman spectroscopy of normal and diseased human breast tissues. Analytical chemistry **1995,** 67 (5), 777-783.

[54] Frushour, B. G.; Koenig, J. L., Raman scattering of collagen, gelatin, and elastin. Biopolymers: Original Research on Biomolecules **1975,** 14 (2), 379-391.

[55] Rygula, A.; Majzner, K.; Marzec, K. M.; Kaczor, A.; Pilarczyk, M.; Baranska, M., Raman spectroscopy of proteins: a review. Journal of Raman Spectroscopy **2013,** 44 (8), 1061-1076.

[56] Dehring, K. A.; Smukler, A. R.; Roessler, B. J.; Morris, M. D., Correlating changes in collagen secondary structure with aging and defective type II collagen by Raman spectroscopy. Applied spectroscopy **2006,** 60 (4), 366-372.

[57] Guilbert, M.; Said, G.; Happillon, T.; Untereiner, V.; Garnotel, R.; Jeannesson, P.; Sockalingum, G. D., Probing non-enzymatic glycation of type I collagen: a novel approach using Raman and infrared biophotonic methods. Biochimica et Biophysica Acta (BBA)-General Subjects **2013,** 1830 (6), 3525-3531.

[58] Brandt, N. N.; Chikishev, A. Y.; Mankova, A. A.; Sakodynskaya, I. K., Effect of thermal denaturation, inhibition, and cleavage of disulfide bonds on the low-frequency Raman and FTIR spectra of chymotrypsin and albumin. Journal of Biomedical Optics **2015,** 20 (5), 051015-051015.

[59] David, C.; Foley, S.; Enescu, M. In Mechanisms of Disulfide Bridges Reduction in Lysozyme Revealed by Raman Spectroscopy and Molecular Computing, AIP Conference Proceedings, American Institute of Physics: 2010; pp 418-419.
